# Internalization and cytotoxicity of graphene oxide and carboxyl graphene nanoplatelets in the human hepatocellular carcinoma cell line Hep G2

**DOI:** 10.1186/1743-8977-10-27

**Published:** 2013-07-12

**Authors:** Tobias Lammel, Paul Boisseaux, Maria-Luisa Fernández-Cruz, José M Navas

**Affiliations:** 1 Departamento de Medio Ambiente, Instituto Nacional de Investigación y Tecnología Agraria y Alimentaria (INIA), Carretera de la Coruña Km 7.5, 28040 Madrid, Spain

**Keywords:** Graphene, Nanoparticle, Cytotoxicity, Uptake, In vitro, Alamarblue, Carboxyfluorescein diacetate acetoxymethyl ester, Neutral red, Oxidative stress, Hep G2

## Abstract

**Background:**

Graphene and graphene derivative nanoplatelets represent a new generation of nanomaterials with unique physico-chemical properties and high potential for use in composite materials and biomedical devices. To date little is known about the impact graphene nanomaterials may have on human health in the case of accidental or intentional exposure. The objective of this study was to assess the cytotoxic potential of graphene nanoplatelets with different surface chemistry towards a human hepatoma cell line, Hep G2, and identify the underlying toxicity targets.

**Methods:**

Graphene oxide (GO) and carboxyl graphene (CXYG) nanoplatelet suspensions were obtained in water and culture medium. Size frequency distribution of the suspensions was determined by means of dynamic light scattering. Height, lateral dimension and shape of the nanoplatelets were determined using atomic force and electron microscopy. Cytotoxicity of GO and CXYG nanoplatelets was assessed in Hep G2 cells using a battery of assays covering different modes of action including alterations of metabolic activity, plasma membrane integrity and lysosomal function. Induction of oxidative stress was assessed by measuring intracellular reactive oxygen species levels. Interaction with the plasma membrane, internalization and intracellular fate of GO and CXYG nanoplatelets was studied by scanning and transmission electron microscopy.

**Results:**

Supplementing culture medium with serum was essential to obtain stable GO and CXYG suspensions. Both graphene derivatives had high affinity for the plasma membrane and caused structural damage of the latter at concentrations as low as 4 μg/ml. The nanoplatelets penetrated through the membrane into the cytosol, where they were concentrated and enclosed in vesicles. GO and CXYG accumulation in the cytosol was accompanied by an increase in intracellular reactive oxygen species (ROS) levels, alterations in cellular ultrastructure and changes in metabolic activity.

**Conclusions:**

GO and CXYG nanoplatelets caused dose- and time-dependent cytotoxicity in Hep G2 cells with plasma membrane damage and induction of oxidative stress being important modes of toxicity. Both graphene derivatives were internalized by Hep G2, a non-phagocytotic cell line. Moreover, they exerted no toxicity when applied at very low concentrations (< 4 μg/ml). GO and CXYG nanoplatelets may therefore represent an attractive material for biomedical applications.

## Background

Graphene is an allotrop of carbon, which consists of a two-dimensional (2D) crystalline lattice of hexagonally arranged, sp^2^-hybridized carbon atoms [1]. Although graphene-like structures have been studied since the middle of the 20th-century [2-4] and free-standing ultrathin graphene sheets have been already imaged in 1962 by Boehm et al. [1], it was not until 2004 that large, single-layered graphene sheets were successfully isolated [5,6]. The ground-breaking experiments by Konstantin Novoselov and Andre Geim, for which they received the 2010 Novel Prize in Physics [7] have paved the way for further fundamental and application-orientated research on this unique material (a “title search” on “graphene” in the ISI Web of Knowledge data base in October 2012 yielded over 19,000 scientific publications since 2005. In comparison, only 84 publications were found for the time before 2005). Multitudinous studies carried out since then revealed that graphene possesses a variety of exceptional physico-chemical properties [8]. Owing to these properties graphene represents an attractive material for a wide range of technical applications. Indeed, the number of filed patents related to graphene has been exponentially increasing over the last years, reaching almost 1000 filed patents by October 2012 [9]. While the research and development of graphene-based electronics is still in its very initial state and the appearance of such electronic devices on the market within the next two decades is rather unlikely [10], the use of graphene in composite materials (similar to those developed using carbon nanotubes) to enhance the latter’s thermal, electric or mechanical properties may not be that far ahead (especially when considering the feasibility of graphene for mass production at low cost) [11]. Many of the composite materials described in patent applications contain platelets of graphene (or oxidized graphene) with lateral dimensions in the nanoscale [12]. Obviously, graphene (or graphene derivative) nanoplatelets permanently embedded in a material matrix pose a low risk to human and environmental health due to low exposure. However, a realistic risk may exist for people working in manufacturing of graphene nano-powder as well as for downstream-users (processing, research and development). Therefore, it is necessary to assess the toxic effects graphene nanoplatelets may exhibit in case of accidental exposure (e.g. through inhalation) [13]. In addition to the broad range of technical applications, nano-sized graphene sheets also attract increasing attention from the biomedical research community [14]. As for other carbon-based nanomaterials such as carbon nanotubes or fullerenes, surface functionalization of the graphene nanoplatelets is a prerequisite for their use in biomedical applications. Two surface-functionalized graphene derivatives, which are used as platform for biomedical applications are graphene oxide (GO) and carboxyl graphene (CXYG) [15]. GO is characterized through the presence of epoxy groups (1–2 ethers) and tertiary hydroxy groups on the basal plane, and lactols, keton, carboxylic acid and ester groups at the edges of the sheet [16-18]. CXYG has a similar chemical structure, but has a higher carboxyl ratio and additionally features ethanoic acid groups (−O-CH_2_-COOH) on sp^3^-hybridized carbon on the basal plane (derived from -OH or -C-OH). Oxidized graphene nanomaterials were demonstrated to be able to serve as efficient carrier systems for the targeted delivery of chemical drugs [19-22] and biomolecules including proteins, DNA and siRNA [23-26] Furthermore, the potential of GO for biosensing [27,28] and bioimaging applications is being explored [29-32]. The feasibility of graphene- and GO-composite materials to enhance the performance of implants and tissue engineering scaffolds is also being investigated [33-36].

Even though the research on technical and biomedical applications of graphene and graphene derivative nanomaterials is expanding rapidly, relatively little is known about their interaction with biological systems or intrinsic toxicity [37]. The sparse literature published on *in vitro* toxicity of graphene nanomaterials suggest that, analogous to other carbon nanomaterials, physico-chemical characteristics may play a critical role in the biological activity of this novel class of nanomaterials [38-40]. Mechanisms that were suggested to underlie the cytotoxic effect include plasma membrane damage [38,41-43], impairment of mitochondrial activity [42,44], induction of oxidative stress [40,42,44,45] and DNA damage [46] eventually leading to apoptotic and/or necrotic cell death [38,42,44,47]. Yet, in some cases, results regarding the cytotoxicity of graphene-based nanomaterials obtained by different authors are conflicting (in particular that for GO). These discrepancies may be due to differences in the intrinsic properties of the nanomaterials tested, the availability of the nanomaterial during the assay or the sensitivity of the cell lines used (among other factors). Furthermore, considering the extremely high specific surface area of graphene nanomaterials and their chemical nature (conjugated π-electron system, presence of reactive functional surface groups), they can be expected to interfere with most of the commonly used bioassay(s) (e.g. physical sorption of assay reagents to the nanomaterial surface, quenching of fluorescent probes, autofluorescence of the nanomaterial). Difficulties in assessing the degree of interference of the tested nanomaterials with the assays employed may have lead to false positive or negative results, and thus could explain some of the inter-study differences detected.

The objective of this study was to evaluate the cytotoxicity and identify the underlying mechanisms of toxicity of two different oxygen-functionalized graphene derivatives, GO and CXYG, using a human hepatoma cell line. As stated above, both graphene derivatives are explored for their use in technical and biomedical applications, so that both accidental and intentional exposure may occur. Moreover, they represent the basic building block of other carbon nanomaterials, such as (hydroxylated and/or carboxylated) fullerenes or carbon nanotubes [10]. The latter have been demonstrated to be subject to chemical and biological degradation yielding breakdown products with hydrodynamic diameters in the submicron range [48-51]. The evaluation of the toxic potential of nano-sized graphene platelets may thus not only contribute to a better understanding of the intrinsic toxicity of engineered graphene nanomaterials, but also of graphene nanoplatelets that could potentially originate from degradation of other graphene-based nanomaterials. A human hepatocellular carcinoma cell line was chosen for performing the experiments, because in the case of exposure (for instance due to accidental inhalation or due to intentional introduction of graphene-based biomedical applications) graphene nanoplatelets may enter the circulatory system and accumulate in the liver [13,52-54]. The Hep G2 cell line was selected in particular because according to a preliminary screening of four hepatoma cell lines (Hep G2, H4IIE, RTH149 and PLHC-1) it was one of the most sensitive for GO and CXYG.

Here we report that GO and CXYG nanoplatelets physically interact with Hep G2 cells and cause plasma membrane damage. Exposure to GO and CXYG was furthermore found to induce oxidative stress and alter metabolic activity and cell ultrastructure. Moreover, we provide new insights into the interaction of graphene nanoplatelets with the plasma membrane, their internalization and intracellular fate.

## Results

### Characterization of GO and CXYG stock suspensions

#### Dynamic light scattering

**Figure 1:**
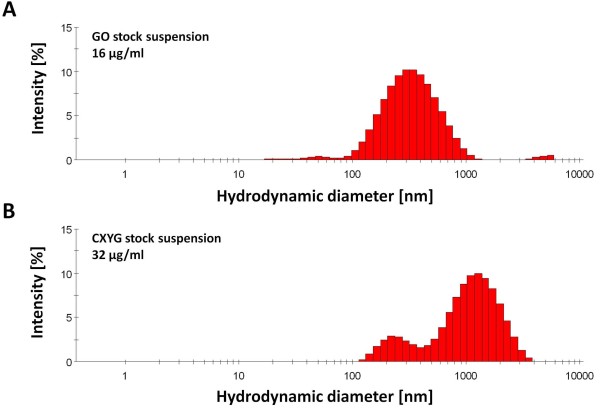
**Size distribution of GO and CXYG in stock suspensions determined by means of DLS.** GO (**A**) and CXYG (**B**) stock suspensions diluted to concentrations of 16 and 32 μg/ml, respectively, were analyzed by means of DLS. The graphs show the average hydrodynamic size distribution by intensity of at least four measurements consecutively conducted on each sample.

GO and CXYG could be readily dispersed in Milli-Q water (at 1 mg/ml) by means of ultrasonication. Yet, DLS and light microscopy analysis indicated that the suspensions contained large aggregates/agglomerates (Additional file 1: Figures S1 and S2, respectively). The aggregates/agglomerates were eliminated by means of centrifugation (Additional file 1: Figures S1 and S2, respectively). The concentration of the supernatants could be successfully estimated using concentration-absorbance standard curves generated from aliquots of the non-centrifuged GO and CXYG suspensions (Curves generated from the non-centrifuged suspensions and curves generated from the supernatant had the same slope) (Additional file 1: Figure S3). Results of DLS measurements performed on these suspensions (in the following text referred to as stock suspensions) met the quality criteria set by the Zetasizer Software. In the GO stock suspension three size populations with average hydrodynamic diameters of 71.4 ± 20, 385.9 ± 18.6 and 4775 nm were identified, with the 385 nm population predominating in terms of intensity (relative intensity: 99.5%) (Figure 1A). In the CXYG stock suspension three size populations with average hydrodynamic diameters of 260.8 ± 24.5, 1110.4 ± 175.3 and 5290.5 ± 107.2 nm could be detected. The relative intensities of the corresponding peaks were 12.6, 85.4 and 2.0%, respectively (Figure 1B).

The ζ-potential of the GO and CXYG stock suspensions was −8.3 mV and −55.1 mV, respectively. Both stock suspensions demonstrated high colloidal stability and could be stored at 4°C for several months without any visible sedimentation or changes in size distribution (Additional file 1: Figure S4).

#### Atomic force microscopy

**Figure 2:**
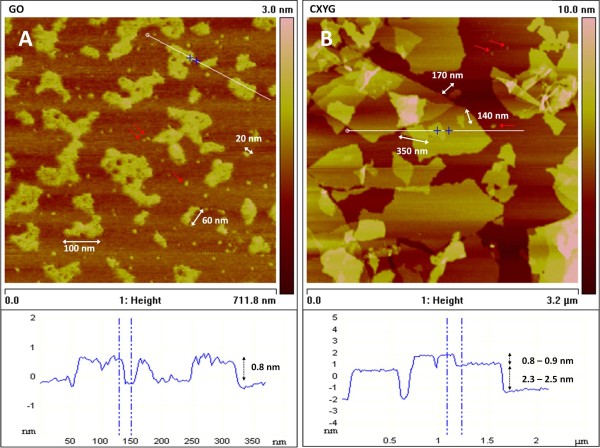
**AFM topographic images of GO and CXYG stock suspensions.** Both stock suspensions comprised nanoplatelets of heterogeneous size and shape. For illustration purposes, the lateral dimension of some GO and CXYG nanoplatelets is displayed in the images (white arrows). Red arrows exemplarily indicate small nanoplatelets whose lateral dimension could not be measured. Height measurements performed on GO stock suspensions (**A**) indicated that GO nanoplatelets were single-layered (~0.8 nm). Height measurements performed on CXYG stock suspensions (**B**) indicated that CXYG nanoplatelets were both single- and few-layered (0.8 - 2.4 nm). Examples of height profiles are presented below the corresponding topographic images (section along the white line visible in the AFM image).

AFM analysis of aliquots of the GO stock suspension revealed that the majority of the imaged GO platelets had a uniform height of 0.8 - 0.9 nm (Figure 2A), i.e. were single-layered [11]. The platelets had rather smooth contours and were of heterogeneous shape. The lateral dimension of the imaged platelets (or aggregates/agglomerates) was of several tens to hundreds of nanometers. A frequency distribution established from size measurements performed on the AFM images (surface area of the individual platelets, three different images, 900 counts) demonstrated that about 95% of the GO platelets had dimensions smaller than 5000 nm^2^, and about 75% had dimensions even smaller than 1000 nm^2^ (Additional file 1: Figure S5) (For illustrative purposes: If the platelets had a disc-like shape, the above stated areas would correspond to platelets with diameters of about 80 and 35 nm, respectively). GO platelets with lateral dimensions of > 1 μm were also observed but sparse (Additional file 1: Figure S6).

In the CXYG stock suspensions, both single-layered and multi-layered platelets were identified. The height of as single-layered interpreted CXYG was ~ 0.8 nm. Most of the multi-layered CXYG had a thickness of ~1.6 or ~2.4 nm, i.e. consisted of two or three carbon layers (Figure 2B). CXYG platelets had sharp-edged contours and were heterogeneous in shape. For CXYG no size distribution could be generated, because CXYG platelets in AFM images were superimposed such that measurements of the area of the individual platelets were not possible. The approximate lateral dimension of the imaged platelets ranged from 100 nm to 1 μm (length) (Figure 2B).

#### Transmission electron microscopy

**Figure 3:**
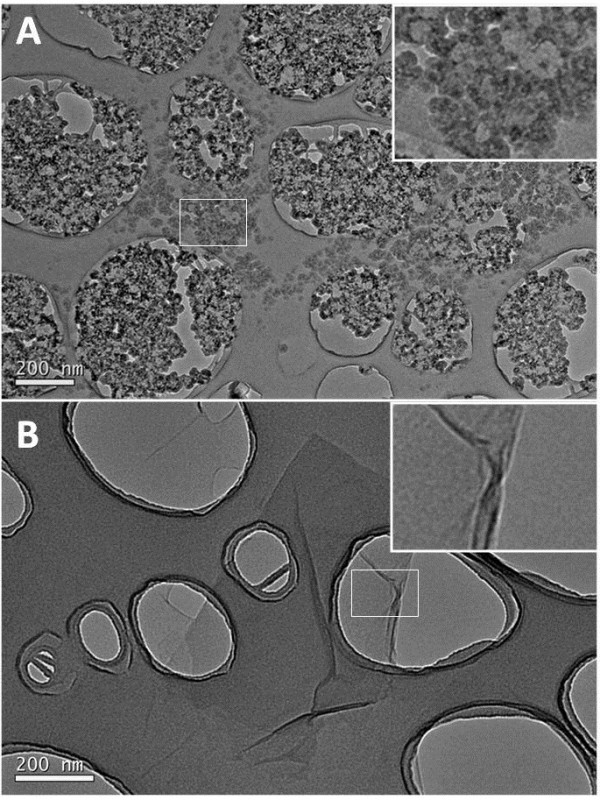
**TEM micrographs of GO and CXYG stock suspensions.** TEM images of GO stock suspensions (**A**) demonstrated aggregates/agglomerates of GO nanoplatelets with lateral dimensions in the lower nanometer range. TEM images of CXYG stock suspensions (**B**) demonstrated comparatively larger, thin sheets. The inserts in the upper right corner of the images show the boxed-in areas at higher magnification. The large round holes visible in both micrographs represent defects in the carbon coating of the copper grid. The scale bar represented in the lower left corner of the images corresponds to 200 nm.

TEM micrographs of the GO stock suspension demonstrated GO nanoplatelets of homogenous, round shape with lateral dimensions in the lower nanometer range (<50 nm). GO nanoplatelets were either present as individual particles or in the form of larger aggregates/agglomerates with lateral dimension ranging from several tens to several hundreds of nanometers of nanometers (Figure 3A). TEM micrographs of CXYG stock suspensions demonstrated CXYG nanoplatelets with lateral dimensions of a few to several hundred nanometers. CXYG platelets also showed fine wrinkles (Figure 3B).

### Characterization of GO and CXYG suspensions in cell culture medium

#### Influence of culture medium composition on colloidal stability of GO and CXYG nanoplatelets

GO and CXYG demonstrated different stability depending on the composition of the cell culture medium (Additional file 2: Figure S1). In the least complex medium (MEM) flocculation of GO and CXYG occurred within the first 5 min. In MEM supplemented with L-Gln and P/S flocculation occurred even almost instantly and was followed by rapid sedimentation of GO and CXYG aggregates/agglomerates. In contrast no flocculation and/or sedimentation were observed in the cell culture medium supplemented with 10% FBS. No major differences were observed regarding the suspension properties of the two graphene derivatives (Additional file 2: Figure S1).

#### Hydrodynamic size distribution and colloidal stability of GO and CXYG suspensions used for cell treatment

DLS analysis of GO and CXYG suspensions in serum-supplemented medium resulted in five intensity peaks, three of which represented nanomaterial populations. The other peaks corresponded to culture medium components. The mean hydrodynamic diameters, the corresponding standard deviations and the relative intensity of all peaks are shown in Tables 1 and 2. The size distribution profiles in form of histograms are represented in Additional file 2: Figures S2 and S3. Note that DLS does not give accurate results for non-spherical particles such as those tested in this study. Yet, it can provide valuable information about changes in the aggregation/agglomeration state of the particles in course of the experiment or dependent on the dilution stage of the suspension. The GO populations detected in the highest concentrated GO sample (16 μg/ml) had an average hydrodynamic diameter of 42.0 ± 11.3, 382.9 ± 22.0 and 4672.5 ± 414.9 nm (Table 1, peak 3, 4 and 5, respectively), i.e. resembled those detected in the GO stock suspension. The hydrodynamic diameter of the identified GO size populations did not change with increasing sample dilution or incubation time (see Table 1 and Additional file 2: Figure S2). The CXYG suspension contained larger platelets (or aggregates/agglomerates) than the GO suspension. The three size populations that were identified in the highest concentrated CXYG sample had an average hydrodynamic diameter of 349.5 ± 42.6, 1805.6 ± 616.8 and 4827.3 ± 497.5 nm (Table 2, peak 3, 4 and 5, respectively). In comparison to the GO suspension, the size distribution profile of the CXYG suspension changed as it was further diluted. Yet, no major changes were observed with increasing incubation time (see Table 2 and Additional file 2: Figure S3). The ζ-potential of the GO and GXYG in serum-supplemented medium was −9.1 mV and −10.2 mV, respectively (the ζ--potential of the medium alone was −0.02 mV).

**Table 1:** Hydrodynamic size distribution in GO suspensions as function of concentration and incubation time

**GO in MEM+**		**Average hydrodynamic diameter**						**Relative intensity**					
**Time**	**Conc.**	**Peak 1**		**Peak 2**	**Peak 3**	**Peak 4**	**Peak 5**	**Peak 1**	**Peak 2**	**Peak 3**	**Peak 4**	**Peak 5**	**PdI**
**[hrs]**	**[μg/ml]**	**d. ± SD [nm]**		**d. ± SD [nm]**	**d. ± SD [nm]**	**d. ± SD [nm]**	**d. ± SD [nm]**	**[%]**	**[%]**	**[%]**	**[%]**	**[%]**	
0	16	4.39	n =1	9.8 ± 2.0	42.0 ± 11.3	382.9 ± 22.0	4672.5 ± 414.9	0.3	9.6	7.4	81.7	1.0	0.7
0	8	1.25	n =1	9.2 ± 2.5	34.4 ± 12.7	314.4 ± 44.0	5140.8 ± 47.3	0.6	13.7	12.2	72.5	1.1	0.9
0	4	-	-	10.0 ± 1.4	35.9 ± 10.3	336.0 ± 38.3	5069.5 ± 163.7	0.0	19.1	17.2	62.7	1.0	1.0
0	2	-	-	9.9 ± 1.2	41.9 ± 7.8	333.8 ± 34.3	4899.4 ± 189.3	0.0	29.6	24.8	44.2	1.7	0.8
48	16	-	-	8.1 ± 1.0	32.5 ± 11.7	367.9 ± 18.6	5081.3 ± 201.5	0.0	7.17	8.1	84.0	0.7	0.7
48	8	-	-	9.5 ± 1.0	36.2 ± 7.1	682.4 ± 96.6	4182.0 ± 514.9	0.0	10.1	7.5	78.8	2.8	1.0
48	4	-	-	9.0 ± 0.4	38.6 ± 5.0	389.0 ± 16.1	-	0.0	19.1	17.1	63.9	0.0	1.0
48	2	-	-	9.6 ± 0.9	37.1 ± 7.6	363.5 ± 50.8	5043.0 n =1	0.0	27.3	21.8	50.6	0.3	0.9
120	16	-	-	9.8 ± 0.6	56.4 ± 19.2	683.7 ± 99.7	4596.3 ± 381.1	0.0	6.2	6.2	85.0	2.7	0.7
120	8	-	-	9.0 ± 1.1	39.1 ± 14.4	346.9 ± 21.2	4796.5 ± 95.5	0.0	8.8	9.1	81.4	0.7	0.7
120	4	-	-	9.8 ± 1.0	33.5 ± 5.5	357.7 ± 33.1	5103.3 ± 145.7	0.0	19.2	14.1	66.2	0.7	1.0
120	2	-	-	10.7 ± 1.2	42.4 ± 9.0	334.2 ± 46.0	4914.7 ± 109.4	0.0	31.3	16.1	51.5	1.2	0.9
blank	-		-	9.6 ± 0.4	62.4 ± 2.5	-	4268.5 ± 129.9	0.0	49.6	46.9	0.0	3.5	0.5

DLS analysis was performed on serial dilutions of a GO suspension (16 μg/ml) prepared in culture medium supplemented with 10% FBS (MEM+). The samples were analyzed directly after preparation as well as after incubation at 37°C for 48 and 120 h, respectively. The average hydrodynamic size is represented as mean diameter (d.) ± standard deviation (SD) in nm. Data are from six consecutively performed measurements. The relative intensities of the size peaks are indicated in [%], respectively. The polydispersity index (PdI) of the analyzed samples is shown in the last column. “n = 1” indicates that no SD could be calculated since the peak was detected in only one of the six performed measurements. The minus (“-“) indicates that no peak could be identified at the respective dilution stage.

**Table 2:** Hydrodynamic size distribution in CXYG suspensions as function of concentration and incubation time

**CYXG in MEM+**		**Average hydrodynamic diameter**						**Relative intensity**					
**Time**	**Conc.**	**Peak 1**		**Peak 2**	**Peak 3**	**Peak 4**	**Peak 5**	**Peak 1**	**Peak 2**	**Peak 3**	**Peak 4**	**Peak 5**	**PdI**
**[hrs]**	**[μg/ml]**	**d. ± SD [nm]**		**d. ± SD [nm]**	**d. ± SD [nm]**	**d. ± SD [nm]**	**d. ± SD [nm]**	**[%]**	**[%]**	**[%]**	**[%]**	**[%]**	
0	32	-	-	-	349.5 ± 42.6	1805.6 ± 616.8	4827.3 ± 497.5	0.0	0.0	15.1	74.1	10.7	0.7
0	16	5.4	n = 1	52.5 ± 9.8	342.5 ± 74.5	2333.8 ± 380.9	4852.5 ± 29.0	0.2	1.4	18.7	74.4	4.3	0.9
0	8	10.2	± 1.9	35.1 ± 9.2	116.2 ± 33.3	796.8 ± 69.3	5449.0 n = 1	9.1	3.6	12.2	74.8	0.4	0.8
0	4	9.7	± 1.4	41.2 ± 9.3	153.1 ± 8.2	821.7 ± 176.3	5516.0 n = 1	17.9	13.2	4.6	64.0	0.3	0.7
48	32	-	-	-	259.3 ± 33.1	1451.3 ± 373.2	5225.5 ± 121.0	0.0	0.0	6.9	89.3	3.7	0.5
48	16	9.7	± 2.1	20.4 ± 1.3	316.2 ± 22.3	1741.7 ± 409.2	4766.0 ± 37.6	2.4	1.3	4.8	87.2	4.4	0.7
48	8	9.0	± 1.4	35.7 ± 8.1	-	896.6 ± 117.5	5134.0 n = 1	9.2	7.5	0.0	83.0	0.3	0.8
48	4	9.2	± 2.3	37.2 ± 11.2	78.4 ± 24.8	735.0 ± 59.0	-	16.0	13.7	0.8	69.6	0.0	0.6
120	32	-	-	69.0 n = 1	283.2 ±164.0	1527.2 ± 483.5	5216.3 ± 286.8	0.0	0.1	14.3	87.7	5.4	0.6
120	16	11.8	± 1.8	-	87.3 ± 21.9	934.3 ± 117.7	-	3.3	0.0	7.1	88.4	0.0	0.6
120	8	9.8	± 2.1	44.4 ± 18.6	-	820.8 ± 40.8	5549.0 n = 1	10.8	7.6	0.0	81.4	0.3	1.0
120	4	10.3	± 0.9	-	107.2 ± 39.0	451.2 ± 83.8	5184.0 ± 116.8	3.2	0.0	14.7	79.9	2.1	0.6
blank		-	-	9.6 ± 0.4	62.4 ± 2.5	-	4268.5 ± 129.9	0.0	49.6	46.9	0.0	3.5	0.5

DLS analysis was performed on serial dilutions of a CXYG suspension (32 μg/ml) prepared in culture medium supplemented with 10% FBS. The samples were analyzed directly after preparation as well as after incubation at 37°C for 48 and 120 h, respectively. The average hydrodynamic size is represented as mean diameter (d.) ± standard deviation (SD) in nm. Data are from six consecutively performed measurements. The relative intensities of the size peaks are indicated in [%], respectively. The polydispersity index (PdI) of the analyzed samples is shown in the last column. “n = 1” indicates that no SD could be calculated since the peak was detected in only one of the six performed measurements. The minus (“-“) indicates that no peak could be identified at the respective dilution stage.

#### Autofluorescence of GO and CXYG

Fluorescence spectra of GO and CXYG stock suspensions (diluted to 10 μg/ml) did not show any significant fluorescence at the excitation/emission wavelengths that were used in the CFDA-AM, ROS (in both assays 485/535 nm), alamarBlue, MMP (in both assays 532/590 nm) and fluorescamine assay (360/450 nm) (Additional file 3: Figure S1). Since emission was only recorded at wavelengths between 250 and 600 nm, no information was obtained if GO or CXYG may fluoresce at the excitation and emission wavelengths used in the NRU assay (532 and 680 nm, respectively). Yet, no autofluorescence was observed in the interference controls that had been included in the assay plate (data not shown).

#### Redox-interaction of GO and CXYG with alamarBlue

No acellular reduction of resazurine was observed upon incubation of the dye (1.25% v/v) with increasing concentrations of GO and CXYG (0.2 – 100 μg/ml) under the normal assay conditions (37°C, 30 min) (Additional file 3: Figure S2).

#### Fluorescence quenching

For all fluorophores (i.e. 5-CF, resorufin and NR) a dose-dependent attenuation of fluorescence intensity was observed (data not shown). The degree of quenching correlated with the logP of the fluorophores suggesting that quenching occurred through sorption of the fluorophores to GO and CXYG. The degree of quenching caused by both graphene derivatives was comparable. At 16 μg/ml GO, 5-CF, resorufin and NR fluorescence was quenched by approximately 5, 9 and 10%. At 16 μg/ml CXYG, 5-CF, resorufin and NR fluorescence was quenched by approximately 3, 8 and 19%, respectively. At 32 μg/ml CXYG 5-CF, resorufin and NR fluorescence was quenched by approximately 7, 16 and 34%, respectively. The degree of quenching was independent of the fluorophore concentration.

### Cytotoxicity of GO and CXYG nanoplatelets

#### AlamarBlue assay

**Figure 4:**
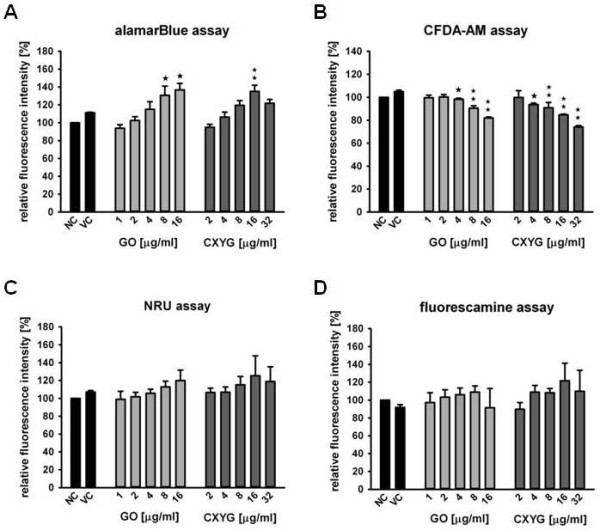
**Effect of GO and CXYG on Hep G2 cell viability.** Hep G2 cells were exposed to increasing concentrations of GO (grey bars) or CXYG (dark grey bars) for 72 h. GO and CXYG suspensions were prepared by diluting aqueous stock suspensions in medium supplemented with 10% FBS. The vehicle control (VC) consisted of 10% (v/v) Milli-Q water in medium. Cells incubated with only medium served as negative control (NC) (black bars). Cytotoxicity of GO and CXYG was assessed by means of the alamarBlue assay (**A**), CFDA-AM assay (**B**), NRU assay (**C**) and the fluorescamine assay (**D**). Bars represent the mean and standard error of the mean (SEM) of at least three independent repetitions. Statistically significant differences with respect to the vehicle control (one-way rmANOVA, Dunnett´s Post-hoc test) are indicated as followed: ^☆^ p < 0.05, ^☆☆^ p < 0.01.

The alamarBlue assay was used to measure the metabolic activity of the cells. Exposure of Hep G2 to GO and CXYG for 72 h resulted in a dose-dependent increase in fluorescence intensity indicating an elevated metabolic activity of these cells (Figure 4A). Although the trend was already discernible at concentrations as low as 2 μg/ml (≈ 0.6 μg/cm^2^), first statistical significant differences with respect to the vehicle control were only detected at concentrations starting from 8 μg/ml (*p* < 0.05) and 16 μg/ml (*p* < 0.01) of GO and CXYG, respectively. In contrast to the observed trend, fluorescence intensity was found to drop again when the exposure concentration reached 32 μg/ml CXYG. No interference of GO and CXYG with the assay could be observed in the controls that were included in the well plate.

#### CFDA-AM assay

The CFDA-AM assay is based on the conversion of CFDA-AM to its fluorescent product 5-CF by cytosolic esterases, which are only retained in cells with intact plasma membrane. In this study a decrease in fluorescence intensity was observed upon 72 h exposure of Hep G2 to increasing GO and CXYG concentrations (Figure 4B). For both graphene derivatives statistically significant differences (*p* < 0.05) compared to the vehicle control were detected at concentrations as low as 4 μg/ml (≈ 1.2 μg/cm^2^). At concentrations > 4 μg/ml means were statistically different at a significance level of *p* < 0.01. Controls that were included in the well plate did not indicate any interference(s) of GO and CXYG with the assay.

#### Neutral red uptake assay

In the neutral red uptake assay, which is based on the accumulation of NR in functional lysosomes, a slight, albeit not statistically significant increase in fluorescence intensity was observed after 72 h exposure to GO and CXYG (Figure 4C). Similar to the results of the alamarBlue assay a decrease in fluorescence intensity was observed at the highest CXYG concentration (32 μg/ml). Controls that were included in the well plate did not indicate any interference with the assay.

#### Fluorescamine assay

Fluorescamine, which reacts with primary amines in proteins, was used to measure the total protein content in treatments and controls. No statistically significant difference (*p* < 0.05) in the total protein content of the different treatments was detected (Figure 4D).

### Generation of intracellular reactive oxygen species

**Figure 5:**
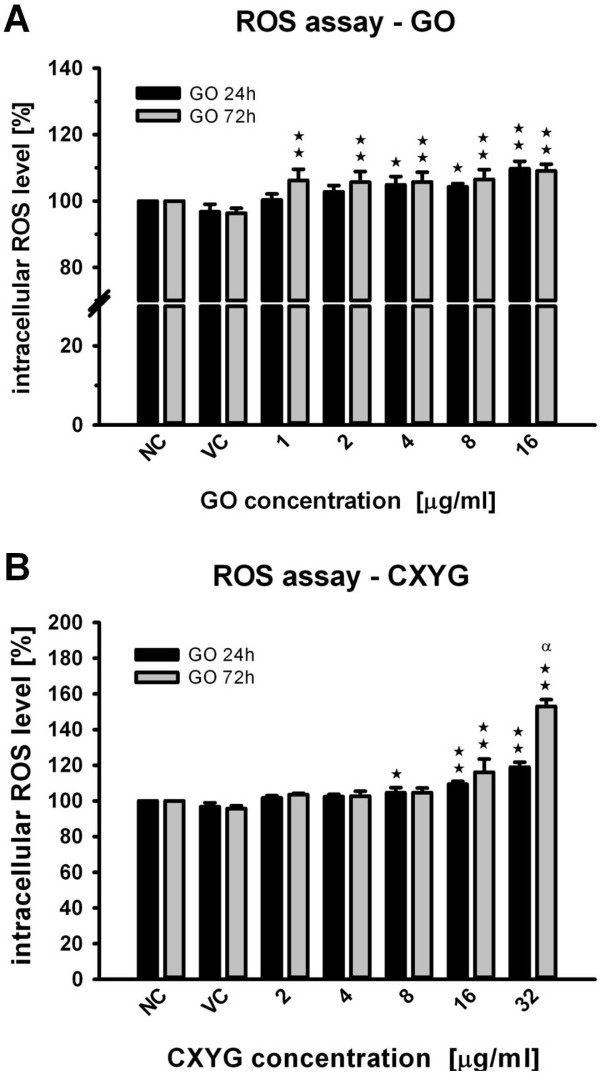
**Level of intracellular reactive oxygen species (ROS) upon exposure to GO and CXYG.** Hep G2 cells were exposed to increasing concentrations of GO (**A**) and CXYG (**B**) for 24 and 72 h (grey and black bars, respectively). Bars represent the mean of at least three independent experiments. Error bars represent the standard error of the mean (SEM). Statistically significant differences with respect to the vehicle control are indicated as ^**☆**^ and ^**☆**☆^ for p < 0.05 and p < 0.01 (One-way rmANOVA, Dunnett’s Post-hoc test), respectively. Statistical significant differences between ROS levels measured for the same concentration at different time points (24 and 72 h) are indicated as α (p < 0.05, t-test).

Both graphene derivatives induced intracellular ROS formation in a dose and time dependent manner (Figure 5). In the 24 h treatment a statistically significant increase with respect to the control was observed at GO concentrations of 4 μg/ml (≈ 1.2 μg/cm^2^) (*p* < 0.05) and higher (*p* < 0.01 for 16 μg/ml GO). After 72 h ROS levels were found to be significantly (*p* < 0.01) elevated at concentrations as low as 1 μg/ml (~110% of the vehicle control) (Figure 5A). In the 24 h CXYG treatment ROS levels were significantly elevated at concentrations of 8 μg/ml (≈ 2.4 μg/cm^2^) (p < 0.05) and higher (p < 0.01). ROS levels at 8 μg/ml did not further increase when incubated for another 48 h (104.6% and 105.1%, after 24 and 72 h, respectively). At higher concentrations (16 and 32 μg/ml, or 4.7 and 9.4 μg/cm^2^, respectively), however, a statistically significant (*p* < 0.01) increase was observed: ROS levels in cells exposed to 32 μg/ml were about 60% higher than those measured in non-treated cells and about 40% higher than those measured at the same concentration after 24 h (Figure 5B).

### Effect of GO and CXYG on mitochondrial membrane potential

**Figure 6:**
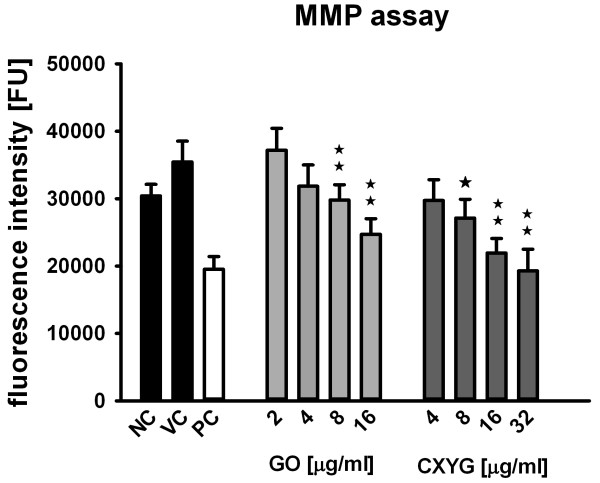
**Mitochondrial membrane potential upon exposure to GO and CXYG.** Hep G2 cells were exposed to increasing concentrations of GO (grey bars) or CXYG (dark grey bars) for 72 h. GO and CXYG suspensions were prepared by diluting aqueous stock suspensions in medium supplemented with 10% FBS. The vehicle control (VC) consisted of 10% (v/v) Milli-Q water in medium. Cells incubated with only medium served as negative control (NC) (black bars). FCCP (20 μM, 10 min) was used as positive control (PC) (white bar). Bars represent the mean and standard error of the mean (SEM) of three independent repetitions. Statistically significant differences with respect to the vehicle control (one-way rmANOVA, Dunnett´s Post-hoc test) are indicated as followed: ^☆^ p < 0.05, ^☆☆^ p < 0.01.

In the MMP-assay statistically significant lower fluorescence intensities were measured in cells that were treated for 72 h with GO or CXYG concentrations ≥ 8 μg/ml (p < 0.05 for 8 μg/ml CXYG, p < 0.01 for concentrations > 8 μg/ml CXYG and p < 0.01 for concentrations ≥ 8 μg/m GO). The fluorescence intensity of the 32 μg/ml CXYG treatment reached levels comparable to those measured in the TCCP positive control (Figure 6).

### Interaction of GO and CXYG nanoplatelets with the cell surface

**Figure 7:**
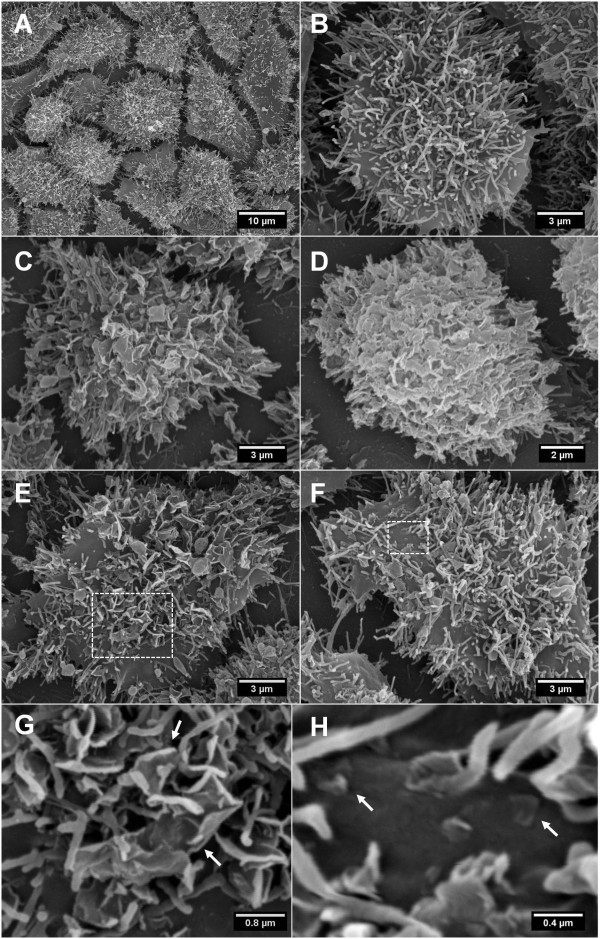
**SEM micrographs of Hep G2 cells after exposure to GO and CXYG for 24 h.** Images **A** and **B** show SEM micrographs of non-treated cells at 1500X and 5000X magnification. The scale bars in these images correspond to 10 and 3 μm, respectively. Control-cells demonstrated healthy cell morphology with numerous microvilli protruding from the cell surface. Images **C** and **D** show SEM micrographs of cells treated with 16 μg/ml GO and 32 μg/ml CXYG, respectively. GO and CXYG platelets deposited and formed a layer completely covering the cell surface. The scale bar displayed in **C** and **D** corresponds to 3 and 2 μm, respectively. Images **E** and **F** show SEM micrographs of cells treated with of 8 μg/ml GO and CXYG, respectively. At this concentration cells were only partly covered with nanomaterial. The scale bars in **E** and **F** correspond to 3 μm. The boxed-in areas (white, dotted line) are shown at higher magnification in images **G** and **F**, respectively. Image **E** shows the interaction of micro-sized GO platelets (white arrows) with microvilli. Image **F** shows the interaction of CXYG nanoplatelets with lateral dimensions between approximately 200 and 400 nm with the plasma membrane (white arrows). The scale bar in **G** and **H** is 0.8 and 0.4 μm, respectively.

Non-treated Hep G2 cells demonstrated numerous microvilli protruding from the cell surface (Figure 7A and B). The surface of cells treated with 16 μg/ml (≈ 4.2 μg/cm^2^) GO and 32 μg/ml (≈ 8.4 μg/cm^2^) CXYG for 24 h were completely covered with GO and CXYG (Figure 7C and D, respectively) (Note: This layer was not discernible in the light microscope). At concentrations of 8 μg/ml and lower only a part of the cell surface was covered with graphene platelets (Figure 7E and F). While the micro-sized platelets were retained by the microvilli, the nano-sized platelets were observed to deposit onto the plane, i.e. microvilli-free plasma membrane domains (Figure 7G and H, respectively). SEM micrographs of cell cultures exposed to high GO and CXYG concentrations for 72 h demonstrated cells with altered cell morphology and an augmented number of apoptotic cells (Additional file 4: Figure S1).

### Interaction with the plasma membrane, internalization and intracellular fate of GO and CXYG nanoplatelets

**Figure 8:**
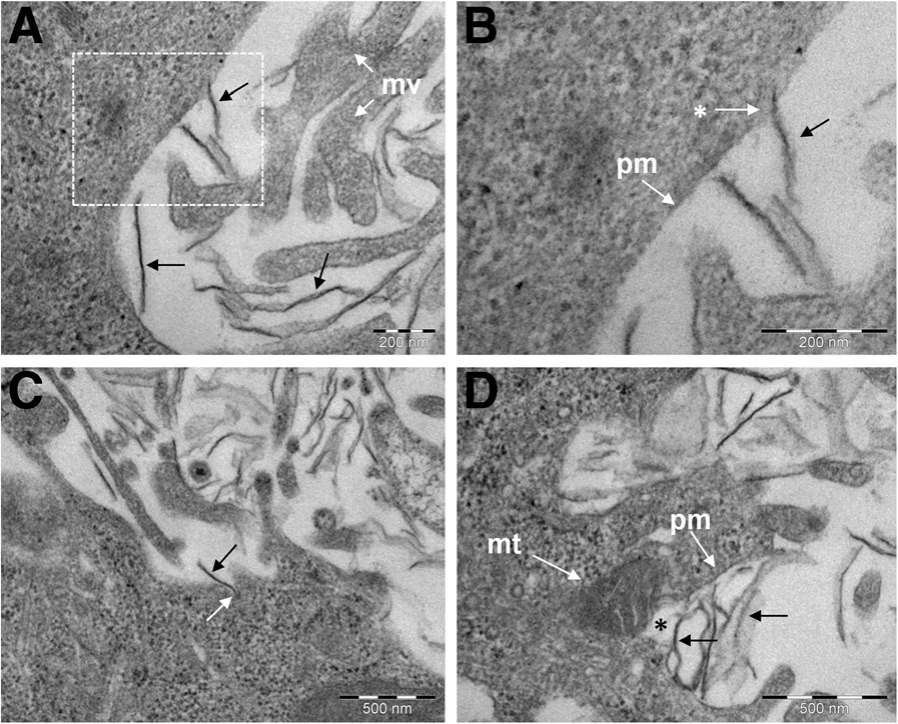
**Interaction of GO nanoplatelets with the plasma membrane of Hep G2 cells. A**) SEM micrograph showing the interaction of GO nanoplatelets (exemplarily indicated by black arrows) with the plasma membrane (pm) and microvilli (mv). **B**) SEM micrographs showing the boxed-in area in **A** at higher magnification. GO nanoplatelet penetrating the plasma membrane (arrow with white asterisk). **C**) Membrane invagination (white arrow) at the site of interaction of a GO nanoplatelet (black arrow) with the plasma membrane. **D**) Disruption of the plasma membrane (black asterisk) at the site of interaction with GO nanoplatelets (exemplarily indicated by black arrows). Scale bars represent 200 nm in **A** and **B**, and 500 nm in **C** and **D**. Black arrows exemplarily indicate GO nanoplatelets. mt: mitochondrion, pm: plasma membrane, mv: microvilli.

**Figure 9:**
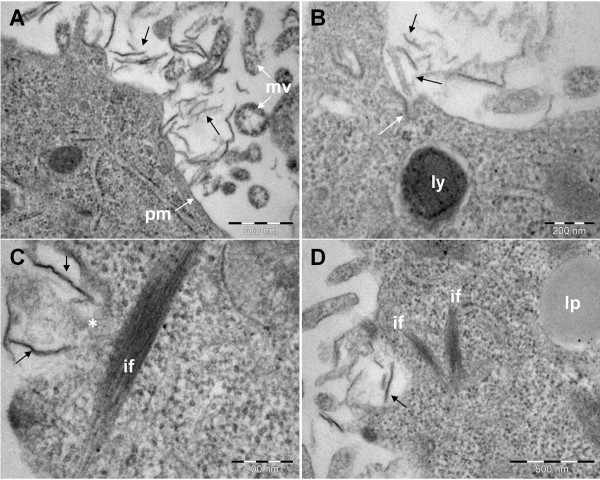
**Interaction CXYG nanoplatelets with the plasma membrane.** (**A**) SEM micrograph showing the interaction of CXYG nanoplatelets (exemplarily indicated by black arrows) with the plasma membrane (pm) and microvilli (mv). (**B**) Vesicle formation (white arrow) at the site of interaction of CXYG nanoplatelets (black arrow) with the plasma membrane. (**C**) and (**D**) CXYG nanoplatelets (black arrows) interacting with the plasma membrane and penetrating the latter (**C**) leading to plasma membrane disruption (the site of disruption is indicated with a white asterisk). Thick intermediate filament bundles (if) are present at the site of injury. Scale bars represent 500 nm in (**A**) and (**D**), and 200 nm in (**B**) and (**C**). Other cellular structures indicated in Figure nine: lp: lipids, ly: lysosomes.

In TEM micrographs of ultrathin sections of GO and CXYG-treated cells numerous nanoplatelets were observed in adjacency to the cell surface (Figures 8 and 9). The nanoplatelets had a lateral dimension of approximately 100 to 300 nm. Nanoplatelets with lateral dimensions bigger than 500 nm were sparse. On some occasions the interaction of the nanoplatelets with the plasma membrane led to the formation of membrane invaginations (Figures 8C and 9B). However no uptake of nanoplatelets into endocytotic vesicles could be observed.

**Figure 10:**
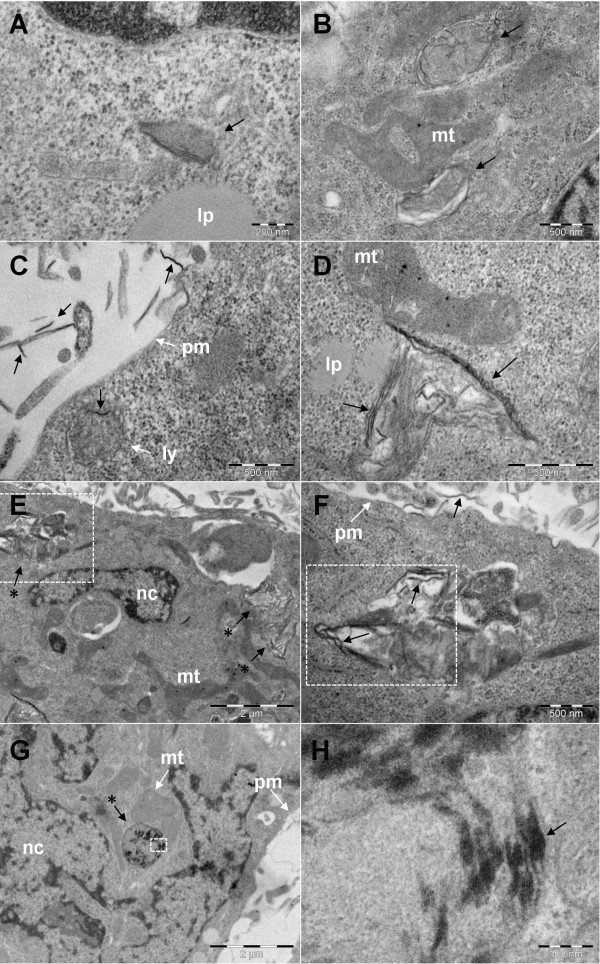
**Intracellular fate of GO nanoplatelets. A**) GO nanoplatelet freely localized (black arrow) in the cytoplasm. **B**) Degraded mitochondria (mt #). **C**) Interaction of a GO nanoplatelet (black arrow) with a lysosome (ly). For comparative purposes extracellular GO nanoplatelets are highlighted (black arrows). **D**) Interaction of cytosolic GO nanoplatelets with a mitochondrion (mt). **E**) Intracellular aggregation of GO nanoplatelets (black arrows with asterisks). **F**) High-magnification image of the boxed-in area in E showing a vesicle with GO nanoplatelet aggregates and degraded cell organelles. GO nanoplatelets (inside and outside the cell) are exemplarily indicated with black arrows. **G**) Phagosome-like vesicle with aggregates of GO nanoplatelets (arrow with asterisk) in close proximity to mitochondria (mt). **H**) High-magnification image (250000X) of the boxed-in area in G showing the laminar nature of the enclosed material (black arrow). Scale bars are 200 nm in **A**, 500 nm in **B**, **C**, **D** and **F**, 2 μm in **E** and **G**, and 100 nm in **H**.

**Figure 11:**
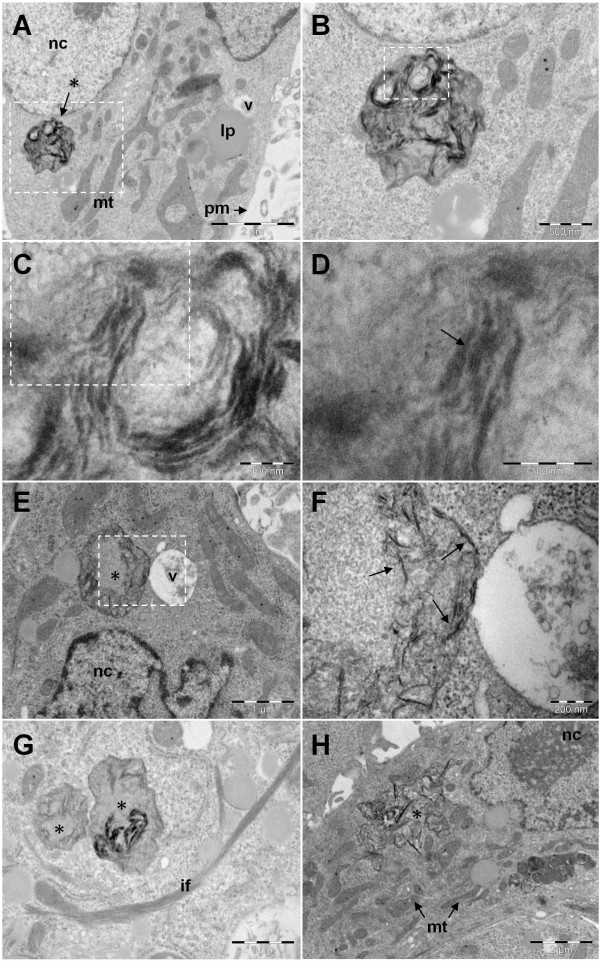
**Intracellular fate of CXYG nanoplatelets.** Images **A**, **B**, **C** and **D** show SEM micrographs of an intracellular CXYG nanoplatelets aggregation (black arrow with asterisk) at different magnifications (50 k, 250 k, 300 k and 400 k X, respectively). The boxed-in areas indicate the part of the image that was amplified in the image of next higher magnification, respectively. Image **E** shows the interaction of an intracellular CXYG aggregation (black asterisk) with a vacuole (v). The site of interaction between the two compartments is represented at higher magnification in **F**. Image **G** shows two intracellular CXYG aggregates (black asterisk) in close proximity to each other and surrounded by intermediate filament bundles (if). **H** shows the ultrastructure of a cell exposed to CXYG for 72 h. The black asterisk indicates the site of CXYG accumulation. The scale bar displayed in **A** and **H** corresponds to 2 μm. The scale bars in **B** and **H** are 500 nm. The scale bars represented in **C** and **D** correspond to 100 nm. The scale bar in **E** and **G** is 1 μm. Other cellular structures indicated in Figure 11 are indicated as followed: pm: plasma membrane, nc: nucleus, mt: mitochondrion, lp: lipid, v: intracellular vacuole.

GO and CXYG nanoplatelets were found to pierce through and mechanically disrupt the plasma membrane (Figures 8B, 9C and D). At some of the sites where the nanoplatelets interacted with or penetrated through the plasma membrane, highly-organized fibrillar structures resembling intermediate filament bundles (compare [55,56]) were observed (Figure 9C and D). TEM images provided evidence that both graphene derivatives crossed the plasma membrane and accumulated inside the cell (Figures 10 and 11). GO and CXYG were present as individual nanoplatelets (Figure 10A) or as aggregate-like structures of different size and compactness (Figure 10D, E and G, Figure 11A, E and G). The aggregates were either freely-localized in the cytosol (Figure 10D) or enveloped within a membrane (Figure 10F and G, Figure 11A, B and E-G) and observed as early as 24 h after cell treatment. TEM micrographs taken at high magnification demonstrated the laminated character of the aggregated material (Figures 10H and 11D). One micrograph showed the fusion of a CXYG-containing vesicle with an intracellular vacuole (Figure 11E and F). Moreover, on some occasions, GO and CXYG aggregates were surrounded by thick intermediate filament bundles (Figure 11G). Interaction of cytosolic nanoplatelets with cell organelles such as lysosomes or mitochondria was also observed (Figure 10D and E, respectively). Furthermore, GO and CXYG-treated cells demonstrated increased mitochondrial calcium accumulation (Figures 10D and 11E), an augmented number of autophagosomes and degraded mitochondria (exemplarily represented in Figure 10B), as well as alterations in chromatin structure.

## Discussion

The cytotoxic potential of GO and CXYG nanoplatelets was assessed in the human hepatoma cell line Hep G2 by means of various cell viability assays based on different toxicity endpoints.

In the CFDA-AM assay both graphene derivatives caused a dose-dependent decrease in fluorescence intensity. According to the assay principle a decrease in fluorescence intensity in the CFDA-AM assay may be indicative of various cytotoxic effects including plasma membrane damage, cell proliferation inhibition and cell death. However, observation in the light microscope and measurement of total protein contents did not disclose any significant differences in the amount of cells in treatments and controls. This suggests that the decrease in fluorescence reflected primarily plasma membrane damage.

Yet, attention must be paid to possible interference of GO and CXYG with the assay. Both graphene derivatives were able to act as fluorescence quenchers. However, considering that the nanomaterial-containing medium was entirely removed prior to adding the probe, the amount of GO and CXYG present at the time of analysis was probably much lower than the concentration at which a relevant degree of quenching was observed. Furthermore, it was observed that despite the high colloidal stability of the suspensions GO and CXYG platelets deposited on the plasma membrane forming a layer, which completely covered the cells’ surface at exposure concentrations ≥ 16 μg/ml (≈ 4.2 μg/cm^2^). The presence of such a layer may locally quench the fluorescence and/or prevent the uptake of CFDA-AM into the cells due to steric, electrostatic or chemical interaction with the probe so that cells would show lower fluorescence intensity, independent of whether or not their plasma membrane is damaged. However, in preliminary experiments in which three different hepatoma cell lines (Hep G2, H4IIE and RTH149) were treated with the same graphene suspension only Hep G2 cells demonstrated a decrease in fluorescence intensity (Additional file 4: Figure S2). This suggests that the observed effect was not due to interference as if this was the case all cell lines would show a similar trend. Moreover, at the lower concentrations at which membrane damage was observed (4 and 8 μg/ml) only a small area of the cell surface was covered with nanomaterial, so that it is unlikely that CFDA-AM diffusion over the cellular membrane was significantly impeded. Yet, the implications for assays that are based on measuring the leakage of cellular macromolecules (e.g. lactate dehydrogenase (LDH) or mRNA) into the medium may be more important than for assays using fluorescent probes of low molecular weight and thus have to be further investigated.

Plasma membrane damage can be the consequence of various cytotoxic effects. The results obtained in this study suggest that the observed loss in plasma membrane structural integrity was associated with a strong physical interaction of GO and CXYG nanoplatelets with the phospholipid bilayer. TEM micrographs of ultrathin sections demonstrated that GO and CXYG nanoplatelets were able to penetrate through the plasma membrane resulting in disruption of the phospholipid bilayer. If the capability of the nanoplatelets to penetrate through the plasma membrane depended on their relative orientation to the latter has to be further examined. Cells responded with the formation of thick intermediate filament bundles, most likely to countervail the tensile forces occurring at the site of interaction/disruption and thus mechanically enforce the plasma membrane and prevent further loss of structural integrity [57-59].

Graphene nanomaterials-caused plasma membrane damage has been reported previously, in both prokaryotic [41,60] and eukaryotic cells [13,38,39,42,43,45]. Liao et al. (2012) demonstrated that both pristine graphene and GO sheets were able to disrupt the plasma membrane of erythrocytes (hemolysis assay).The EC_50_ calculated for the hemolytic activity of GO platelets with similar dimensions to those used in our study was 30.5 μg/ml (after 3 h of incubation with agitation) [43]. Chang et al. (2010), on the contrary, were not able to observe any adverse effect of GO nanoplatelets with lateral dimensions of about 200, 400 and 800 nm on plasma membrane integrity in the human lung cell line A459. In fact, at exposure concentrations ≥ 50 μg/ml the LDH activity was observed to be even lower than in the control [45]. In a study by Sasidharan et al. (2011), in which carboxyl-functionalized graphene was compared with pristine graphene, no LDH leakage could be observed neither - even at concentrations as high as 300 μg/ml [38]. Zhang et al. (2010) observed that graphene aggregates/agglomerates that had sedimented onto the surface of rat PC12 cells caused an increase in LDH leakage only at the highest exposure concentration (100 μg/ml) [42]. These findings are partly conflicting with those obtained in our study. However, the LDH assay may not be the most appropriate one to assess the membrane disrupting potential of graphene nanomaterials. First, as discussed above, a graphene layer covering the cell surface may impede leakage of LDH into the medium. And second, any enzyme “successfully” released into the medium may adsorb to the suspended nanomaterial and thus be inactivated.

The observed effect of GO and CXYG on plasma membrane integrity was congruent with the concentration-dependent increase in alamarBlue reduction that was observed upon exposure. Although the exact mechanism through which alamarBlue is reduced still has to be elucidated, it is generally assumed that reduction occurs in the mitochondria. There, due to its relatively less negative redox potential, it can receive electrons from various components of the electron transport chain including NADPH, NADH, FADH2, FMNH2, and cytochromes [61]. As a consequence, an increase in resazurine reduction could be indicative of an increase in the metabolic activity of the cells and/or an increase in cell number [61]. Since no significant differences in the protein content of treated and not-treated wells were observed, the increased resazurine reduction could well be related with an augmented metabolic activity of the individual cells. The inverse correlation of metabolic activity and degree of membrane damage may suggest that cells have initiated energy-dependent processes involved in plasma membrane repair (e.g. rearrangement of cytoskeletal elements, biosynthesis of proteins and lipids, trafficking of exocytotic vesicles to injured sites at the plasma membrane) [62].

The increase in fluorescence observed in the alamarBlue assay could also be due to interference or autofluorescence properties of the graphene derivatives used in this study. However, neither acellular alamarBlue reduction by GO and CXYG nor autofluorescence at the excitation and emission wavelength used could be observed. Besides, incubation of resorufin (=the fluorescent reduction product of alamarBlue) with increasing concentrations of GO and CXYG demonstrated that both graphene derivatives were able to quench its fluorescence. Thus, interference of the tested nanomaterials with the assay would rather lead to an underestimation of the signal.

It must also be kept in mind that resazurine can be reduced by mechanisms different to those stated above. Gonzalez and Tarloff (2001), for example, demonstrated that resazurine can be reduced by cytosolic and microsomal enzymes (S9-fraction) [63]. Thus, an increased expression/activity of the latter, as it is for example observed during detoxification, could also explain the increase in fluorescence intensity. Besides, Lancaster et al. (1996) suggested that resazurine reduction may occur through scavenging of electrons from lipid peroxidation cascades in dying cells [61,64] and Prutz et al. (1996) demonstrated that resazurine reduction may occur through reaction with free radicals [65]. Since in our study resazurine reduction was correlated with intracellular ROS levels, these mechanisms could also explain the elevated fluorescence intensity at high exposure concentrations.

Induction of oxidative stress is considered one of the principal mechanisms underlying nanomaterial toxicity [66,67]. In our study, GO and CXYG nanoplatelets were observed to induce the generation of intracellular ROS in a concentration and time-dependent manner. In addition, GO and CXYG-induced ROS formation seemed to follow different kinetics. For GO, maximum ROS levels were reached after exposure to 16 μg/ml for 24 h. In cells treated with lower GO concentrations (1 – 8 μg/ml) intracellular ROS levels kept increasing in the lapse between 24 and 72 h and eventually reached levels comparable to those measured at 16 μg/ml. On the contrary, exposure to low concentrations of CXYG (< 8 μg/ml) did not result in significantly increased ROS levels (not even upon exposure for 72 h). Yet, ROS levels in cells treated with high CXYG concentrations (≥ 8 μg/ml) were observed to increase considerably in the lapse between 24 and 72 h.

Regarding the oxidant-generating potential of GO, the obtained results are consistent with those reported by other authors. As in the present study, Yuan et al. (2012) could not detect any significant increase in intracellular ROS levels in Hep G2 cells exposed to 1 μg/ml of single-layered GO for periods of less than 24 h [47]. Yet, the results presented here demonstrate that exposure to such low concentrations can indeed lead to intracellular ROS formation in this cell line if the exposure duration exceeds 24 h. The ability of GO to induce the generation of intracellular ROS was also assessed in other cell lines. A549 cells exposed to 10 μg/ml GO for 24 h demonstrated comparable ROS levels to those determined in this study [45]. In human skin fibroblasts, however, no significant increase with respect to the control could be detected after 24 h of exposure to concentrations as high as 25 μg/ml [43]. The discrepancy between the results obtained in this study and those stated above (including ours) might be due to differences in the lateral size of the platelets tested (> 1 μm), the suspension protocol (serum-free medium), the assay protocol (loading of the cells with the dye DCFH-DA was carried out prior to treatment) or the sensitivity of the cell line. To our knowledge, no data on the oxidant-generating ability of CXYG have been reported in the scientific literature to this day.

The fact that GO and CXYG-induced ROS generation displayed different kinetics suggests that the underlying ROS-generating mechanisms are distinct. The exact mechanism(s) through which a nanomaterial exerts oxidative stress is relatively difficult to identify and still remains to be elucidated for most nanomaterials including graphene and graphene derivative nanoplatelets. An integrative consideration of results obtained by different assays, however, can help to get a first indication about the possible mechanisms involved. In general, it is distinguished between direct and indirect mechanisms of ROS generation. Direct ROS generation typically involves processes that are independent of the presence of biological systems (acellular ROS generation), i.e. are solely a function of the nanomaterial’s physico-chemical properties. Indirect ROS generation, on the contrary, typically involves cellular (i.e. biochemical) processes that were triggered by the nanomaterial beforehand [68]. In non-inflammatory cells, one of the probably most important nanomaterial-triggered mechanisms leading to increased intracellular ROS formation is impairment of mitochondrial function. To assess whether or not the increased ROS levels may have originated from GO- and CXYG-induced alterations in mitochondrial processes, the nanomaterials´ effect on the mitochondrial integrity was investigated. It was observed that exposure to GO and CXYG nanoplatelets resulted in a decrease in fluorescence intensity in the MMP assay indicating mitochondrial membrane depolarization and/or a decrease in the amount of (functional) mitochondria. These findings are consistent with those of Li et al. (2012), who reported that the MMP decreased in a dose and time-dependent manner in the macrophage cell line RAW 264.7 exposed to increasing concentrations of pristine graphene [44]. Depolarization of the mitochondrial membrane can be due to the loss of both structural and functional integrity of the mitochondrion [69]. Mitochondrial dysfunction is known to be associated with oxidative damage of mitochondrial macromolecules including mtDNA, lipids and proteins caused by reaction with intracellular ROS [69]. Structural damage of mitochondria can be provoked directly, i.e. by physical interaction of the nanomaterial with the mitochondrial membrane [70] or indirectly, e.g. by physically disrupting the membrane of other cell organelles, such as lysosomes, resulting in release of hydrolytic enzymes into the cytosol [71].

Both mechanisms would require prior internalization of the nanomaterial, which in the present study was observed. In a few cases interaction of the nanoplatelets with the plasma membrane was observed to be attended by invagination of the latter. Dutta et al. (2007) demonstrated that serum albumin adsorbed to the surface of carbon nanotubes facilitates their uptake via scavenger receptor-mediated endocytosis [72]. Interaction of scavenger receptors in the plasma membrane of Hep G2 with serum proteins adsorbed to the surface of GO and CXYG nanoplatelets may explain the observed membrane invagination at the site of platelet/membrane interaction. However, in this study no evidence for successful uptake of GO or CXYG into endocytotic vesicles was found. TEM micrographs demonstrated that GO and CXYG nanoplatelets were able to penetrate through the plasma membrane and were freely localized in the cytosol. Besides, TEM images showed aggregates of different size and compactness, whereas most (but not all) were enveloped by intracellular membranes. This suggests that GO and CXYG nanoplatelets that entered the cytosol were recognized by the cell as foreign particle, concentrated in one or more defined areas in the cytosol and then packed into intracellular vesicles to isolate the nanomaterial and protect itself from further damage.

Yet, in an initial phase of the internalization process GO and CXYG nanoplatelets and aggregates were freely localized in the cytosol and thus potentially able to directly interact with cellular organelles including mitochondria and lysosomes. In the present work no direct interaction of individual nanoplatelets with lysosomes could be observed. In addition, no adverse effect of GO and CXYG nanoplatelets on lysosomal function was detected in the NRU assay. All these results together suggest that GO and CXYG nanoplatelet-induced ROS generation and mitochondrial damage were not related with release of lysosomal iron or hydrolytic enzymes into the cytosol.

Direct interaction of GO and CXYG nanoplatelets with mitochondria could be observed in one micrograph. Moreover, GO and CXYG-treated cells demonstrated an augmented number of autophagosomes, in some of which degraded mitochondria could be identified. Degraded mitochondria could also be observed in the cytosol, i.e. not yet enclosed in autophagocytotic vesicles. Thus, it may be possible that enhanced intracellular ROS levels originated from mitochondrial damage. If mitochondrial damage was caused by physical interaction of GO and CXYG nanoplatelets with the mitochondrial membrane or is a secondary effect of a possible oxidative damage of mitochondrial macromolecules due to elevated intracellular ROS levels remains to be elucidated [70,73].

So far, uptake of graphene nanomaterials has been almost exclusively reported for phagocytotic cells [13,40,44]. To our knowledge, there is only one published study reporting accumulation of a graphene nanomaterial in the cytosol of a non-phagocytotic cell line [38]. Yue et al. (2012), who studied the uptake of GO in four hepatoma cell lines including Hep G2 could not observe any internalization. They suggested that the negative surface charge of GO may have led to electrostatic repulsion of the platelets from the plasma membrane [40]. The SEM micrographs in the present study however demonstrate that GO and CXYG have a rather high affinity to biological membranes. These findings suggest that other physico-chemical properties may determine whether graphene nanomaterials are internalized and that further research has to be carried out into this direction.

## Conclusions

**Figure 12:**
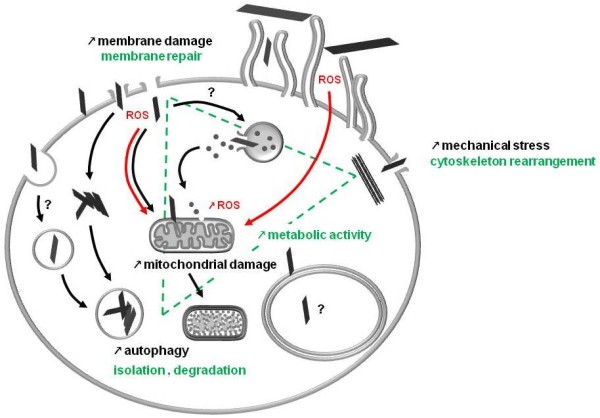
**Hypothetic model of graphene nanomaterial internalization and cytotoxicity.** GO and CXYG nanoplatelets penetrate through the plasma membrane into the cytosol, are concentrated and encapsulated in intracellular vesicles. Cells respond with the formation of cytokeratin filament bundles to mechanically reinforce the plasma membrane and initiate plasma membrane repair mechanisms. These processes involve an increase in metabolic activity. Exposure to GO and CXYG nanoplatelets results in elevated intracellular ROS levels, perturbation of mitochondrial structure and function, and an augmented number of autophagosomes.

In this study it was demonstrated that ultrasonication followed by a centrifugation step yields stable stock suspensions. The suspension protocol may be used to produce stock suspension of other commercially available graphene derivatives similar to the ones used in this study. For cell culture experiments serum-supplemented medium should be used as in serum-free medium rapid flocculation and sedimentation of the nanomaterial occurs. Yet, as demonstrated by the SEM micrographs, also when working with stable suspensions graphene nanoplatelets may still adsorb onto the cell surface. In the present study it was observed that cells exposed to GO and CXYG concentrations of 16 μg/ml for 24 h were completely covered with nanomaterial. Thus, when assessing the cytotoxicity of graphene nanomaterials the exposure concentrations should not exceed those used in this study as the local exposure concentration is already maximal. Any further increase in the concentration may cause nanomaterial-unspecific cell damage due to mechanical stress and/or increase the probability of interference of the nanomaterial with the assay. In this study, first cytotoxic effects were observed at concentrations as low as 4 μg/ml. Among the modes of action assessed, plasma membrane damage and induction of oxidative stress appeared to play a crucial role. Moreover, GO and CXYG nanoplatelets were able to pierce through the plasma membrane and enter the cytosol, Cells were able to successfully isolate the nanomaterial by enclosing it in intracellular vesicles (a summary illustration with a hypothetic model of graphene nanomaterial internalization and cytotoxicity is shown in Figure 12). Graphene derivative nanomaterials may thus represent an attractive platform for biomedical applications such as drug carriers. Yet, due to the sparse *in vitro* and *in vivo* toxicological data and indications that graphene nanoplatelets may accumulate in various organs including lungs, liver, spleen and kidney and may not easily be cleared from the body [74], further research has to be carried out to identify the physico-chemical properties that determine their toxicity and ways to enhance its biocompatibility and performance for use in biomedical applications.

## Methods

### Nanomaterials

Single-layer Graphene oxide (GO) and Carboxyl graphene (CXYG) were purchased from ACS Material, LLC. (Ames, IA, USA). GO was prepared by modified Hummers´s method and exfoliated as described in Allister et al. (2007) and supplied as thin film with a single layer ratio of ~ 99% [75]. The weight content in carbon and oxygen was 51.26% and 40.78%, respectively (C/O ratio: 1.67). CXYG was supplied as dry powder. The weight content in carbon and oxygen was 50.36% and 42.23%, respectively (C/O ratio: 1.59). The carboxyl ratio was 5%. Both graphene derivatives had a purity of approximately 99%.

#### Chemicals and cell culture products

Cell culture: Ultraglutamine 1 (200 mM) (L-Gln), fetal bovine serum (FBS), penicillin and streptomycin (P/S) (10 000 U/ml / 10 mg/ml), non-essential amino acids (NEAA) 100X, Trypsin EDTA (200 mg/l EDTA, 17 000U trypsin/L), cell culture EMEM (Eagle’s Minimum Essential Medium) was sourced from Lonza (Barcelona, ES). Phenol red-free Minimum Essential Medium (MEM) was purchased from PAN-Biotech (Aidenbach, DE).

Assay reagents and chemicals: AlamarBlue and 5-carboxyfluorescein diacetate, acetoxy methyl ester (CFDA-AM) were purchased from Life Technologies (Madrid, ES). Neutral red (3-amino-7-dimethylamino-2-methylphenanzine hydrochloride) solution (0.33%), 6-carboxy-2′7′-dichlorofluorescein diacetate (DCFH-DA), 5-carboxyfluorescein (5-CF), resorufin, fluorescamine, chloramine-T trihydrate, sodium dodecyl sulfate (SDS) and bovine serum albumin (BSA) were sourced from Sigma-Aldrich (Madrid, ES).

Solvents, fixatives and resin: 2-propanol, acetonitrile, acetone and glacial acetic acid were purchased from Sigma-Aldrich (Madrid, ES). Ethanol was from Panreac (Barcelona, ES). Paraformaldehyde (16%) was sourced from Electron Microscopy Sciences (Hatfiled, UK), glutaraldehyde (25%), osmium tetroxide (2%) and Spurr’s resin were purchased from TAAB Laboratories Equipment Ltd (Aldermaston, UK). High grade purity water (> 18 MΩ cm-1) was obtained from a Milli-Q Element A10 Century (Millipore Iberia, ES).

### Characterization

#### Preparation of GO and CXYG stock suspensions

GO and CXYG stock suspensions were prepared in sterile, 10 ml Pyrex glass tubes (SciLabware Ltd., Stone, UK). First, GO and CXYG were dispersed in sterile Milli-Q water at a concentration of 1 mg/ml by means of ultrasonication at 37 kHz for 2 h in a 4.25 L bath-type ultrasonic cleaner unit (Elmasonic S 40/(H), Elma GmbH & Co. KG, Singen, DE). Following sonication the suspensions were centrifuged at 1300 g for 30 min (Lince R, Orto Alresa, Madrid, ES) and the supernatants transferred to a fresh Pyrex glass tube. The concentration of the supernatants was estimated by means of a concentration-absorbance standard curve generated from aliquots of the original, i.e. not centrifuged stock suspensions. Serial dilutions and absorbance measurements were performed in 96-well plates and a microplate reader (Tecan Genios, Tecan Group Ltd., Männedorf, CH) equipped with a 340 nm filter, respectively. The concentration of the centrifuged CXYG suspension was adjusted to 320 μg/ml. The concentration of the GO suspension was adjusted to the next lower dilution stage (160 μg/ml) as its concentration was below 320 μg/ml after centrifugation.

#### Dynamic light scattering

The hydrodynamic size of GO and CXYG in both the stock suspension and the culture medium was determined by means of dynamic light scattering (DLS) analysis using a Zetasizer Nano-ZS apparatus (Malvern Instruments Ltd., Malvern, UK). Milli-Q water and culture medium were used as background controls. At least four measurements were taken of each sample. The number of runs per measurement, the attenuator and the optimal measurement position were automatically determined. Data were analyzed using Zetasizer Software version 6.34 (Malvern Instruments Ltd.).

#### ζ-Potential measurements

ζ-potential measurements were performed using disposable capillary cuvettes (Malvern Instruments Ltd., UK) and a Zetasizer Nano-ZS apparatus (Malvern Instruments Ltd., Malvern UK). Four measurements were taken of each sample. The number of runs was set automatically.

#### Atomic force microscopy

Aliquots (25 μl) of diluted GO and CXYG stock suspensions (final concentration: 12.5 - 25 μg/ml) were transferred onto freshly exfoliated mica substrates and air-dried at room temperature overnight. Atomic force microscopy (AFM) imaging of the dried samples were performed in tapping mode on a Nanoscope IIIa Mulitmode apparatus (Veeco, Plainview, NY, USA) using a TESP-SS cantilever with a tip radius of 2 nm and a spring constant of 42 N · m^-1^ (Veeco). Height and phase images were recorded simultaneously. Images were analyzed using NanoScope software version 6.24r1 (Veeco). Measurements of the lateral dimension of the nanoplatelets were performed on AFM topographical images converted to 8-bit format and tresholded using ImageJ version 1.34u (Wayne Rasband, National Institutes of Health, USA).

#### Transmission electron microscopy

Transmission electron microscopy (TEM) was performed to morphologically characterize the GO and CXYG platelets dispersed in the stock suspensions. Carbon-coated copper grids were drop-coated with the stock suspensions. TEM analysis was carried out using a JEOL 2100 HT (JEOL Ltd., Tokyo, JP) operated at an accelerating voltage of 200 kV.

#### Preparation of GO and CXYG suspensions in culture medium

For cell culture studies the aqueous stock suspensions had to be diluted in culture medium. The composition of the culture medium is known to have an important influence on the colloidal stability and biological activity of nanomaterials. Therefore it was investigated how the medium supplements influence the stability of GO and CXYG. For this purpose, GO and CXYG stock suspensions were diluted 1:10 in three different complex culture media: MEM, MEM supplemented with 1% L-Gln and 1% P/S, and MEM supplemented with 1% L-Gln, 1% P/S and 10% FBS, respectively. The stability of the suspension was assessed by means of DLS and visual observation.

#### Routine cell culture

The human hepatocellular carcinoma cell line Hep G2 was obtained from the American Type Culture Collection (ATTC) (Manassas, VA, USA). Hep G2 cells were cultured in 75 cm^2^ Cell Star cell culture flasks (Greiner Bio-One GmbH, Frickenhausen, DE) in EMEM supplemented with 1% NEAA, 1% P/S, 1% ultraglutamine and 10% FBS. The flasks were incubated at 37°C in a humidified 5% CO_2_ atmosphere and split twice a week using PBS/EDTA and trypsin.

### Cytotoxicity

The toxicity of graphene oxide (GO) and carboxyl graphene (CXYG) nanoplatelets towards the human hepatoma cell line Hep G2 was assessed using various cytotoxicity assays based on different toxicological endpoints (metabolic activity, cell membrane disruption, lysosomal integrity, total protein content). The seeding and exposure protocol was similar for all 96-well plate-based cytotoxicity assays (CFDA-AM assay, alamarBlue assay, neutral red uptake (NRU) assay, fluorescamine assay).

#### Exposure

Hep G2 cells were seeded into transparent, flat-bottom 96-well plates (growth area: 0.34 cm^2^) (Greiner Bio-One GmbH, Frickenhausen, DE) by adding 100 μl of cell suspension (7.5 × 10^5^ cells/ml) to each well. After seeding the well plates were incubated at 37°C in a humidified 5% CO_2_ atmosphere for 24 h. GO and CXYG stock suspensions (160 and 320 μg/ml, respectively) were diluted 1:10 in phenol red-free MEM supplemented with 1% L-Gln, 1% P/S and 10% FBS (in the following text referred to as MEM+) and applied to the cell culture plate in which serial dilutions (dilution factor of 2) were performed. As a positive control, a subset of wells was treated with increasing concentrations of SDS (0.02 mM – 0.5 mM, dilution factor 1.5). Cells treated with phenol red-free MEM + served as negative control. Cells treated with 10% (v/v) Milli-Q water/phenol red-free MEM + served as vehicle control. The microwell plates were incubated at 37°C in a humidified CO_2_ atmosphere for 72 h and then subject to analysis.

#### AlamarBlue, CFDA-AM and Neutral Red Uptake assay

The alamarBlue, CFDA-AM and NRU assay were performed on the same set of cells. The assays were conducted following a modified version of the protocol described by Dayeh et al. (2005) [76]. Prior to adding the probes the exposure medium was removed and the cells rinsed twice with 200 μl PBS. Then, 100 μl serum- and phenol red-free MEM containing 1.25% (v/v) alamarBlue and 4 μM CFDA-AM was added to each well. The 96-well plates were incubated for 30 min in the dark at 37°C and 5% CO_2_. Subsequently the fluorescence intensity was measured at excitation and emission wavelengths of 532 and 590 nm (resorufin) or 485 and 535 nm (5-CF), respectively, using a microplate reader (Tecan Genios, Tecan Group Ltd., Männedorf, CH). Subsequently, the medium was removed and the cells were washed once with PBS. 100 μl of NR solution (0.03 mg/ml in phenol red-free MEM) were added per well and the plates incubated for 1 h in the dark at 37°C in a humidified 5% CO_2_ atmosphere. After the incubation period the NR solution was removed, the cells rinsed twice with 200 μl PBS and the NR retained in the cells extracted with an acidified (1% glacial acetic acid) 50% ethanol/49% Milli-Q water solution (150 μl/well). NR fluorescence was measured at 532 nm/680 nm (excitation/emission) using a microplate reader (Tecan Genios). The fluorescent values were corrected for the cell-free control and normalized against the medium control.

#### Fluorescamine assay

After exposure, the GO and CXYG-containing medium was discarded and the cells rinsed twice with PBS. The well plates were immediately frozen using liquid nitrogen and then stored at −20°C for 1 h. Then, 75 μl of PBS and 75 μl of fluorescamine solution (0.15 mg/ml fluorescamine in acetonitrile) was added to each well and the plates placed on a horizontal shaker for 15 min in the dark. Subsequently, the fluorescence intensity was measured using a Tecan Genios microplate reader equipped with a 360 nm excitation and 450 nm emission filter, respectively. The fluorescent values were corrected for the cell-free control and normalized against the medium control.

### Interference

In the cytotoxicity assays two different interference controls were included. To assess if GO and CXYG adsorbed to the polystyrene surface of the culture well may interfere with the assay cell-free wells were treated with the highest exposure concentration. Non-treated cell-free wells served as reference. To assess if GO and CXYG adsorbed to the cell monolayer may interfere with or contribute to the fluorescence signal intensity at the excitation and emission wavelengths used, cells were treated with the highest exposure concentration but no probe was added at time of analysis. Non-treated cells to which no dye was added served as reference. In addition, prior to performing the cytotoxicity assays, it was assessed if the tested nanomaterials interfere with the assay reagents or their fluorescent conversion products (see below).

#### Autofluorescence

Due to their aromatic nature graphene and graphene derivatives are principally able to exhibit fluorescence. To assess if the tested graphene derivatives show autofluorescence and thus may interfere with the fluorescence-based cytotoxicity assays used in this study three-dimensional fluorescence spectra of GO and CXYG stock suspensions (10 μg/ml) were recorded using a Perkin-Elmer (Norwalk, CT) LS 55 luminescence spectrometer. Fluorescence emission was measured over a wavelength range from 250 to 600 nm. The excitation wavelength was sequentially increased from 250 to 600 nm by 5 nm steps. Data were visualized in form of excitation − emission matrix plots using the software WinLab 4.00.02 (Perkin-Elmer, Inc., 2001, Norwalk, CT, USA).

#### Redox-interaction of GO and CXYG with assay reagents

Resazurine (7-Hydroxy-3H-phenoxazin-3-one 10-oxide), the active compound in the commercial solution alamarBlue, is a redox-sensitive dye and thus able to undergo redox (reduction-oxidation) reactions with GO and CXYG. To assess if GO or CXYG residues retained in the culture vessel can reduce resazurine also acellularly, and thus lead to an increase in fluorescence intensity independent of the metabolic activity or number of cells, alamarBlue (1.5% (v/v) prepared in phenol red-free medium without FBS) was incubated with increasing concentrations of GO and CXYG (0.2 – 100 μg/ml) at 37°C for 30 min (cp. alamarBlue assay protocol). Subsequently the well plates were read at 532 nm/590 nm (= excitation and emission maxima of resorufin - the conversion product of alamarBlue) in a Tecan Genios microplate reader.

#### Fluorescence quenching

Fluorescence quenching was assessed by incubating the fluorophores that are formed in course of the CFDA-AM , alamarBlue and neutral red (NR) uptake assay (i.e. 5-carboxyfluorescein (5-CF), resorufin and protonated NR) with increasing concentrations of GO and CXYG (0.2 – 100 μg/ml), respectively. The used fluorophore concentrations corresponded to the maximal concentration that can be expected to be formed in the respective assays (4 μM 5-CF, 1 μM resorufin, 0.03 mg/ml NR) and to 10% of the maximal concentration that can be expected in the assay (0.4 μM 5-CF, 0.1 μM resorufin), respectively. 5-CF and resorufin were prepared in serum- and phenol red-free medium (cp. CFDA-AM and alamarBlue assay protocol). NR was diluted in 50% ethanol, 49% Milli-Q water, 1% glacial acetic acid (= extraction solution used in the NRU assay). The assay itself was carried out as followed: GO or CXYG stock suspensions (non-centrifuged) (0.1 mg/ml Milli-Q) were serially diluted (1:2) in the respective fluorophore solutions. The serial dilutions were performed in a 96-well plate. Fluorescence intensity was measured in a Tecan Genios microplate reader using 532 nm/590 nm, 485 nm/535 nm, and 532 nm/680 nm excitation and emission filters for resorufin, 5-CF and NR, respectively.

### Generation of reactive oxygen species

Cell seeding and treatment with GO and CXYG was performed as describe above. Cells treated with increasing concentrations of chloramine-T trihydrate (0.04 mM – 10 mM) were used as positive control. After treatment, the microwell plates were incubated at 37°C and 5% CO_2_ for 24 or 72 h, thereupon the cells were rinsed twice with PBS prewarmed to 37°C (150 μl/well). Then, 100 μl of a 20 μM DCFH-DA solution prepared in serum- and phenol red-free MEM (=loading solution) was added to each well and incubated for 15 min in the dark at 37°C and 5% CO_2_. The loading solution was discarded and cells were washed twice with 150 μl PBS. The increase in fluorescence intensity was measured over 90 min in 15 min intervals in a Tecan Genios microplate reader using 485 nm and 530 nm excitation and emission filters, respectively. Relative reactive oxygen species (ROS) levels were calculated as followed:

$$\left[[\left(t_{i\;\mathrm{treatment}}/t_{0\;\mathrm{treatment}}\times 100\right)‒\left((,t_{i\;\mathrm{negative}\;\mathrm{control}}/t_{0\;\mathrm{negative}\;\mathrm{control}}\times 100\right)\right]]\times 100$$

with “i” being the time at which no further increase in fluorescence intensity was observed (typically after 60 min).

### Mitochondrial membrane potential

The mitochondrial membrane potential (MMP) was measured using the positively-charged fluorescent dye TMRE (tetramethylrhodamine, ethyl ester), which readily accumulates in active mitochondria. TMRE was purchased as part of the TMRE Mitochondrial Membrane Potential Assay Kit (Abcam, Cambridge, UK), which also included FCCP (carbonyl cyanide 4-(trifluoromethoxy)phenylhydrazone) – an ionophore uncoupler of oxidative phosphorylation that served as positive control. The assay was performed according to a protocol developed on basis of the assay procedure provided by the manufacturer. Hep G2 cells were seeded into μClear bottom, black 96-well plates (growth area 0.34 cm^2^) (Greiner Bio-One GmbH, DE) (7.5 × 10^4^ cells/well) and treated with GO and CXYG as described in the previous sections. After 72 h of exposure at 37°C and 5% CO_2_ a subset of wells was treated with 20 μM FCCP (100 μl/well). The nanomaterials-containing exposure medium was replaced with medium (100 μl/well). Thereupon, the cells were incubated for another 10 min at 37°C and then stained with TMRE prepared in phenol-red MEM + (final concentration in the well: 0.5 μM). After the staining period (15 min at 37°C in the dark) the dye was aspirated and rinsed twice with 0.2% BSA in PBS. Subsequently the fluorescence intensity was measured using a Tecan Genios microplate reader equipped with at 532 and 590 nm excitation and emission filters, respectively.

### Scanning and transmission electron microscopy of biological samples

Hep G2 cells were seeded onto poly-L-lysine coated glass coverslips (BD biosciences, Erembodegem, BE) located in the wells of a 24-well plate (growth area: 1.9 cm^2^) (Greiner Bio-One GmbH, DE). The cell number at time of seeding was 2.0 × 10^5^ cells/well. The medium volume was 0.5 ml. The plates were incubated overnight at 37°C and 5% CO_2_ and thereafter exposed to 16 μg/ml GO and 32 μg/ml CXYG for 24, 48 or 72 h. Cells exposed to only medium and 10% Milli-Q water were used as negative and vehicle control, respectively. After the exposure period, the nanomaterial containing medium was removed and the cells washed three times with Millonig buffer (pH 7.3) and then fixed with 4% paraformaldehyde and 2.5% glutaraldehyde in Millonig buffer (pH 7.3). After fixing, the cells were washed twice and stored in Millonig buffer over night at 4°C. The following day, the cells were postfixed in 1% osmium tetroxide in bidestilled water for 1 h. The cells were rinsed three times with bidestilled water and subsequently dehydrated in an increasing acetone gradient (30, 50, 70, 80, 90, 95 and 100% (2×), sequentially applied in 15 min steps). For scanning electron microscopy (SEM), following dehydration, the samples were critical point-dried, metalized and analyzed in a JOEL 6400 JSM scanning electron microscope (JEOL Ltd., Tokyo, JP) operated at 35 kV. For TEM, following dehydration, cells were infiltrated with Spurr´s resin (Spurr´s resin : acetone (1:3) for 1 h, Spurr´s resin : acetone (1:1) for 1 h, Spurr´s resin : acetone (3:1) for 2 h, 100% Spurr´s resin overnight). The following day, the coverslips with the cell monolayer were embedded in fresh Spurr´s resin, which was then left to polymerize at 65°C for 48 h. Afterwards the coverslip was removed from the cured resin block by immersing the latter in liquid nitrogen. Subsequently ultrathin sections were prepared by cutting the resin block in the plane of the cell monolayer using a Leica Ultracut E ultramicrotome (Leica Microsystems, Wetzlar, DE) equipped with a diamante knife. Ultrathin sections were stained with 1% uranyl acetate in bidestilled water followed by Reynolds’ lead citrate and then analyzed in a JOEL 1010 JEM transmission electron microscope (JEOL Ltd, Tokyo, JP) operated at 100 kV.

### Statistical analysis

Results of cytotoxicity, ROS and MMP assays represent the means and standard errors (SEM) of at least three independent experiments, in which each treatment was applied in triplicate. Statistical analysis was performed using Sigma Plot version 12.0 (Jandel Scientific, San Rafael, CA, USA). Significant differences among treatments were determined by one-way repeated measures analysis of variance (rmANOVA, *p* < 0.05). All data were tested beforehand for normality (Shapiro-Wilk test, *p* < 0.05) and equal variance (*p* < 0.05). Significant differences between treatments and the control were determined by applying a Dunnett’s Post hoc test. For comparisons of two groups a Student´s t-test was performed.

## Competing interests

The authors declare that they have no competing interests.

## Authors’ contributions

TL was responsible for conception and design of the study, acquisition, analysis and interpretation of all data and drafting of the manuscript. PB contributed to the acquisition and analysis of cytotoxicity and DLS data. MLFC has been involved in conception and design of the experiments. JMN suggested to carry out this work and has been involved in conception and design of the study, and in drafting and critical revision of the manuscript. All authors read and approved the final manuscript.

## Authors’ information

TL is an Early Stage Researcher (ESR) within the Marie Curie Initial Training Network Environmental ChemoInformatics (ITN-ECO) sponsored by the Seventh Framework Programme of the European Union. He has graduated in Biology with specialization in Cell Biology, Biochemistry, Genetics and Ecotoxicology from Goethe-University Frankfurt am Main, DE. His PhD work at the National Institute for Agricultural and Food Research and Technology (INIA), Madrid, Spain focuses on mechanisms of toxic action of nanomaterials.

PB is a Marie Curie Short-term fellow within the Marie Curie Initial Training Network Environmental ChemoInformatics (ITN-ECO) sponsored by the Seventh Framework Programme of the European Union. He obtained his Master degree in “Health Engineering” with speciality “Environmental Project Management” from the University of Montpellier.

MLFC is researcher at National Institute for Agricultural and Food Research and Technology (INIA) in Madrid. She has a wide expertise in the field of toxicology and environmental toxicology where she has published studies related with bioaccumulation, mechanisms of toxicity and with the fate of pesticides in vegetables. Currently, at the INIA Department of Environment, she is more focus on the study of the effects of nanoparticles and endocrine disruptors *in vitro* and *in vivo*. She has also been implied in the risk assessment evaluation for the human and environmental health of pesticides, biocides and contaminants.

JMN holds a position as research scientist at the Spanish National Institute for Agricultural and Food Research and Technology (INIA). Since 2008 he is the Director of the Department of Environment of INIA. He graduated as Biologist at the University of Salamanca (1991) and obtained his PhD degree at the University of Valencia (1997). He has worked in Germany and France for several years and has obtained different grants and contracts in competitive calls. His research interests are related with the mechanisms of toxic action of endocrine disrupters and nanoparticles.

## Supplementary Material

Additional file 1: Figure S1 Hydrodynamic size distribution of ultrasonicated GO suspensions in Milli-Q water before and after centrifugation. DLS measurements performed on the non-centrifuged dispersions demonstrated low inter-measurement reproducibility (A). DLS measurements performed on the supernatants of the centrifuged dispersion demonstrated good inter-measurement reproducibility (B). **Figure S2.** Light microscopy images of Hep G2 cells treated with non-centrifuged and centrifuged GO suspensions. Cells were incubated for 24 h with the suspensions, washed twice with PBS and then analyzed in a Zeiss Axiovert 25 inverted microscope (100X magnification). Cell cultures treated with suspensions (100 μg/ml) prepared from the non-centrifuged stock suspensions were covered with large aggregates/agglomerates (arrow) (A). In cell cultures treated with suspensions (16 μg/ml) prepared from the centrifuged stock suspensions no aggregates/agglomerates were discernible. **Figure S3.** Estimation of the concentration of GO and CXYG stock suspensions. A) Standard curves generated from the non-centrifuged suspensions. B) Absorbance values of the corresponding supernatants and serial dilutions thereof plotted against the concentrations estimated using the standard curves shown in A. The slope of the curve derived from the non-centrifuged suspension was similar to the slope of the curve derived from the centrifuged suspension indicating that the agglomeration state of the suspensions had no influence on their absorptivity. **Figure S4.** Photograph of GO and CXYG stock suspensions after eight weeks storage at 4°C. GO and CXYG stock suspensions demonstrated high colloidal stability. No sedimentation of GO or GXYG could be observed. **Figure S5.** Size distribution of platelets in the GO stock suspension established on the basis of surface area measurements in AFM topographical images. **Figure S6.** AFM topographical image of the GO stock solution. In addition to GO nanoplatelets with lateral dimensions in the lower nanometer range (cp. Results, Figure 2A), few platelets with sizes from several hundred nanometers to a few micrometers were identified. Click here for file

Additional file 2: Figure S1 Influence of culture medium composition on colloidal stability of GO and CXYG nanoplatelets. GO and CXYG stock suspensions were diluted 1:10 in three different complex cell culture media (A and B, respectively): MEM, MEM supplemented with 1% L-Gln and 1% P/S, and MEM supplemented with 1% L-Gln, 1% P/S and 10% FBS. The photographs were taken 10 minutes after preparation of the samples. Medium supplementation with FBS was essential to obtain dispersion with high colloidal stability. The presence of L-Gln and P/S did accelerate GO and CXYG nanoplatelet flocculation and sedimentation. **Figure S2.** Hydrodynamic size distribution in GO suspensions as function of concentration and incubation time. DLS analysis was performed on serial dilutions of a GO suspensions prepared in serum-supplemented culture medium (16 μg/ml). The samples were analyzed directly after preparation and after incubation at 37°C for 48 and 120 h, respectively. No significant change in the size distribution profile was observed as function of sample concentration or incubation time. **Figure S3.** Hydrodynamic size distribution in CXYG suspensions as function of concentration and incubation time. DLS analysis was performed on serial dilutions of a CXYG dispersion prepared in serum-supplemented culture medium (32 μg/ml). The samples were analyzed directly after preparation and after incubation at 37°C for 48 and 120 h, respectively. No significant change in the size distribution profile was observed as function of sample concentration or incubation time. **Figure S4.** Hydrodynamic size distribution profile of serum-supplemented MEM. Click here for file

Additional file 3: Figure S1 Three-dimensional (3D) fluorescence spectra of GO and CXYG stock suspensions. A) 3D fluorescence spectrum of a 10 μg/ml GO/Milli-Q water dispersion B) 3D fluorescence spectrum of a 10 μg/ml CXYG/Milli-Q water dispersions. C) 3D fluorescence spectrum of Milli-Q water (blank). **Figure S2.** Redox-reaction of alamarBlue with GO and CXYG in absence of cells. AlamarBlue dissolved in phenol red-free medium without FBS was incubated with inreasing concentrations of GO and CXYG (0.2 – 100 μg/ml) at 37°C for 30 min. No reduction of alamarBlue was observed. Click here for file

Additional file 4: Figure S1 SEM micrographs of Hep G2 cells exposed to 16 and 32 μg/ml GO and CXYG for 72. Image A shows a SEM micrograph of untreated cells. Image B shows cells treated with 32 μg/ml CXYG. Image C shows cells treated with 16 μg/ml GO. The boxed-in area is shown at higher magnification in image D. Scale bares are 30 μm in A and B, 50 μm in C and 4 μm in D. White arrows exemplarily indicate apoptotic cells (communication with Dr. Covadonga Alonso, Departamento de Biotecnología, Instituto Nacional de Investigación y Tecnología Agraria y Alimentaria, INIA) being detached from the substrate and neighboring cells, and characterized by a round cell shape and plasma membrane blebbing. **Figure S2.** Differential cytotoxicity of GO and CXYG. Click here for file
