# Supplementary material for: Internalization and cytotoxicity of graphene oxide and carboxyl graphene nanoplatelets in the human hepatocellular carcinoma cell line Hep G2
Source: Part Fibre Toxicol. 2013 Jul 12;10:27. doi: 10.1186/1743-8977-10-27 (PMC3734190; doi:10.1186/1743-8977-10-27)
Supplement: Additional file 1: Figure S1 — Hydrodynamic size distribution of ultrasonicated GO suspensions in Milli-Q water before and after centrifugation. DLS measurements performed on the non-centrifuged dispersions demonstrated low inter-measurement reproducibility (A). DLS measurements performed on the supernatants of the centrifuged dispersion demonstrated good inter-measurement reproducibility (B). Figure S2. Light microscopy images of Hep G2 cells treated with non-centrifuged and centrifuged GO suspensions. Cells were incubated for 24 h with the suspensions, washed twice with PBS and then analyzed in a Zeiss Axiovert 25 inverted microscope (100X magnification). Cell cultures treated with suspensions (100 μg/ml) prepared from the non-centrifuged stock suspensions were covered with large aggregates/agglomerates (arrow) (A). In cell cultures treated with suspensions (16 μg/ml) prepared from the centrifuged stock suspensions no aggregates/agglomerates were discernible. Figure S3. Estimation of the concentration of GO and CXYG stock suspensions. A) Standard curves generated from the non-centrifuged suspensions. B) Absorbance values of the corresponding supernatants and serial dilutions thereof plotted against the concentrations estimated using the standard curves shown in A. The slope of the curve derived from the non-centrifuged suspension was similar to the slope of the curve derived from the centrifuged suspension indicating that the agglomeration state of the suspensions had no influence on their absorptivity. Figure S4. Photograph of GO and CXYG stock suspensions after eight weeks storage at 4°C. GO and CXYG stock suspensions demonstrated high colloidal stability. No sedimentation of GO or GXYG could be observed. Figure S5. Size distribution of platelets in the GO stock suspension established on the basis of surface area measurements in AFM topographical images. Figure S6. AFM topographical image of the GO stock solution. In addition to GO nanoplatelets with lateral dimensions in the lower nanometer [file 1743-8977-10-27-S1.pdf]

Additional file 1

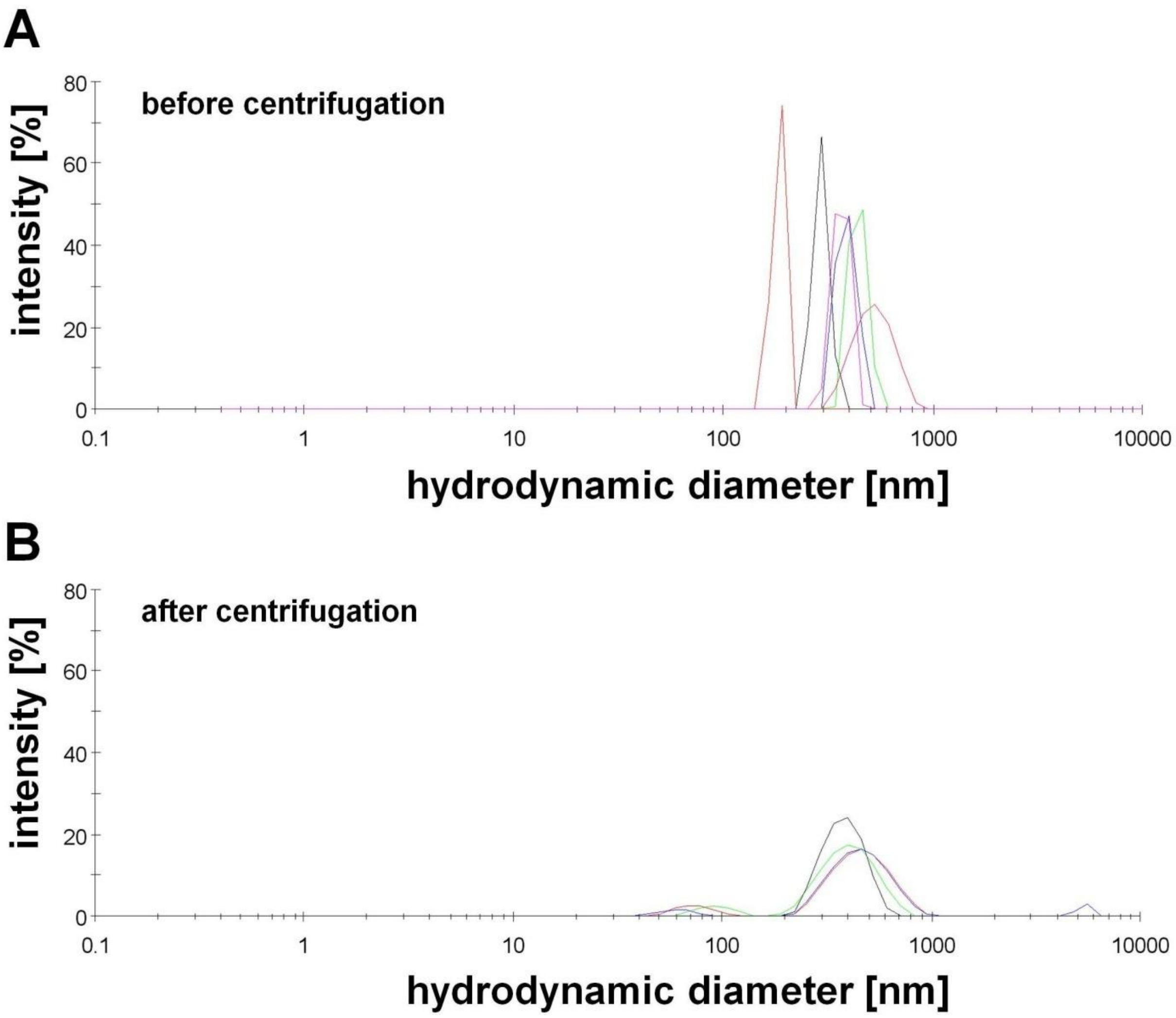

Figure 1: Size distribution of ultrasonicated GO suspensions in Milli-Q water before and after centrifugation determined by means of DLS. GO was dispersed in Milli-Q water at a concentration of 1 mg/ml using ultrasonication. DLS measurements performed on aliquots of the dispersion demonstrated low inter-measurement reproducibility (A). Consequently, the GO dispersion was centrifuged to eliminate not fully exfoliated material and large aggregates/agglomerates. DLS measurements performed on the supernatants of the centrifuged dispersion demonstrated good inter-measurement reproducibility (B).

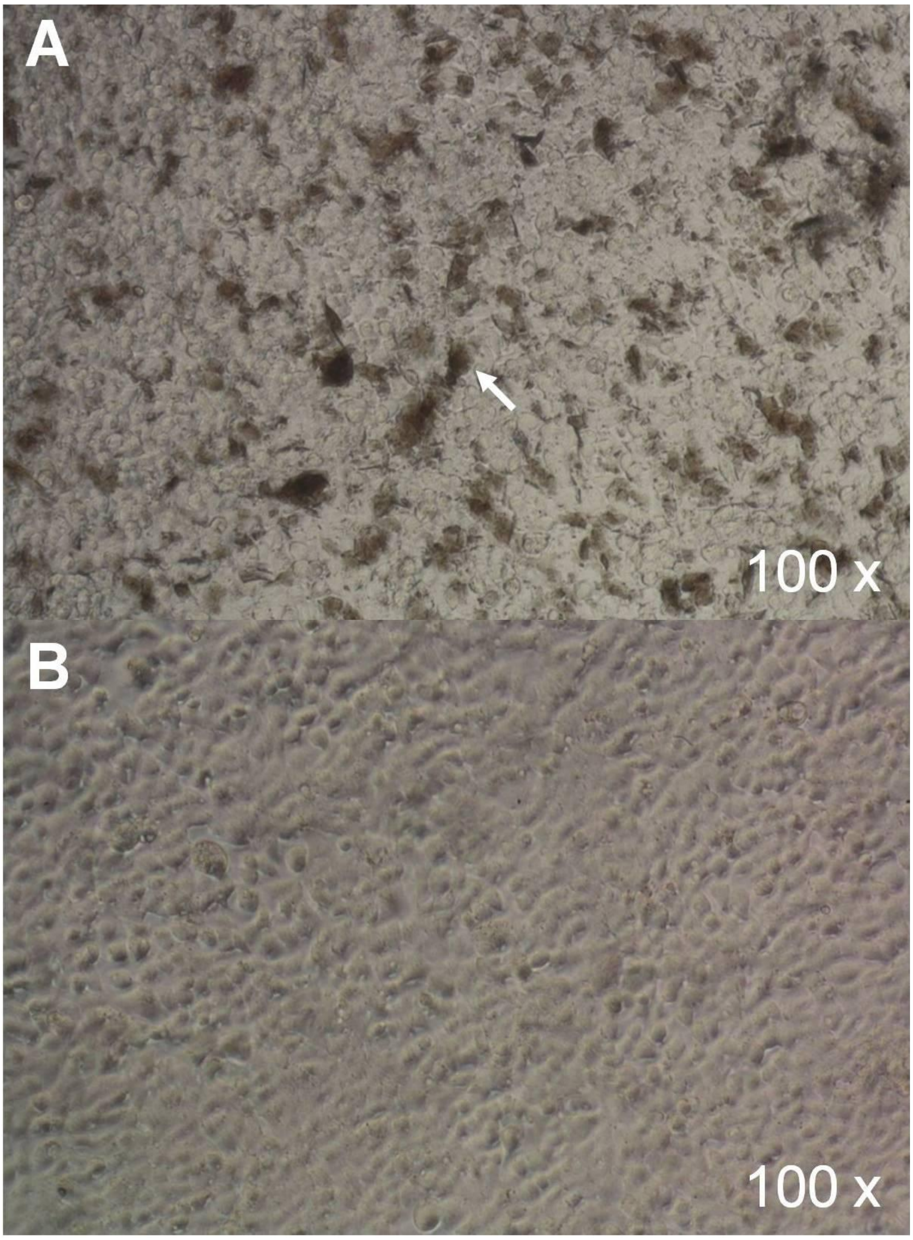

Figure 2: Light microscopy images of Hep G2 cells treated with non-centrifuged and centrifuged GO suspensions. A) Hep G2 cells treated with a 100 µg/ml GO suspension prepared from the non-centrifuged stock suspension. B) Hep G2 cells treated with a 16 µg GO suspension prepared from the centrifuged stock suspension (1300 g, 30 min). After 24 h the microwells were washed twice with PBS and then analysed in the light microscope. In A large particles (aggregates/agglomerates/not exfoliated material) were observed in the wells on top of the cell monolayer (white arrow). In B no GO aggregates/agglomerates were discernible under the light microscope. Both images were taken at 100X magnification using a Zeiss Axiovert 25 inverted microscope (Carl Zeiss Microscopy GmbH, Jena, DE).

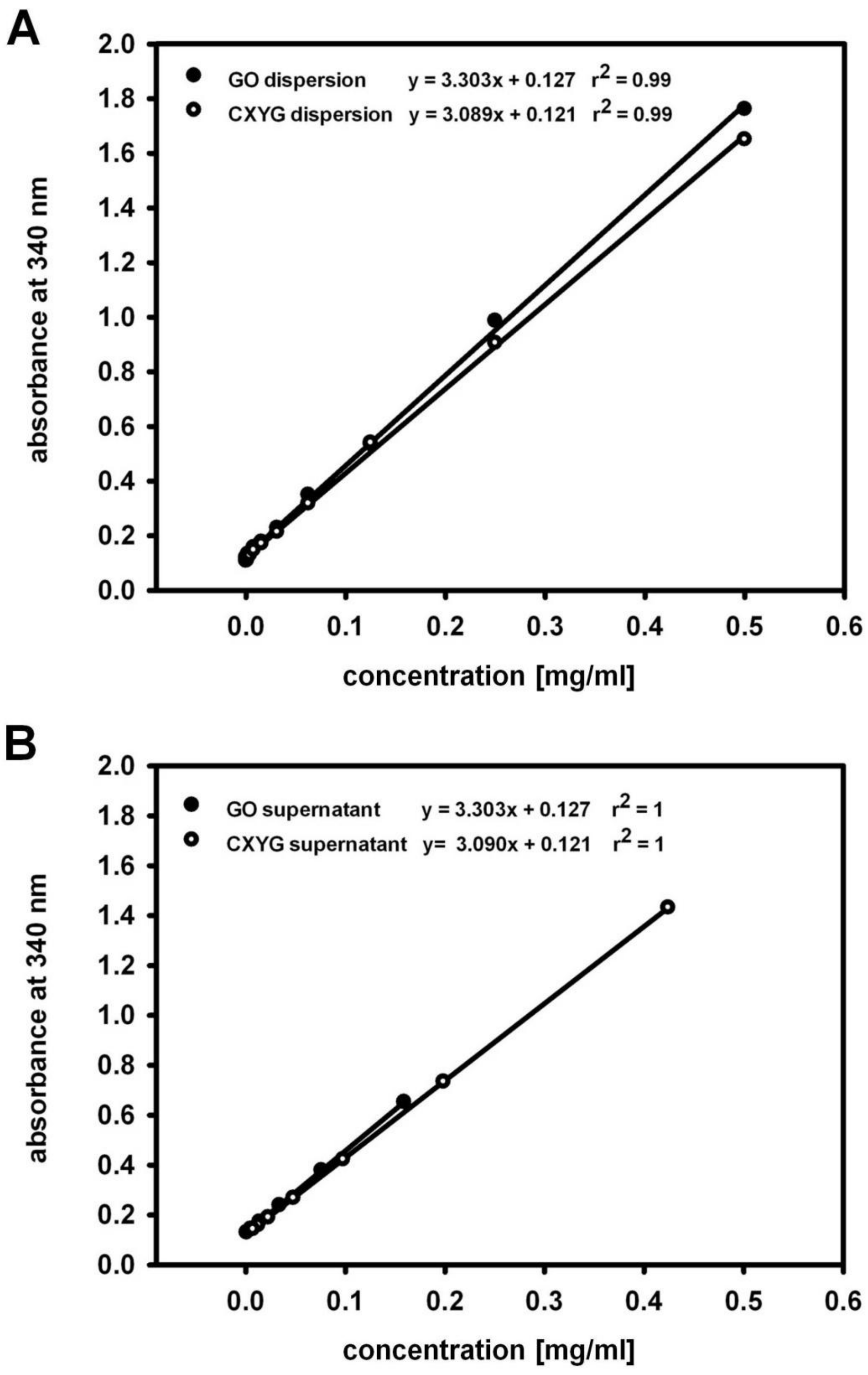

Figure 3: Estimation of the concentration of GO and CXYG stock suspension. A) Standard curves generated from the non-centrifuged GO and CXYG suspensions. B) Absorbance values of the corresponding supernatants and serial dilutions thereof plotted against the concentrations estimated by means of the standard curves shown in A. Note that the slope of the curve derived from the non-centrifuged suspension was similar to the slope of the curve derived from the centrifuged suspension indicating that the concentration and agglomeration state of the suspensions had no influence on their absorptivity.

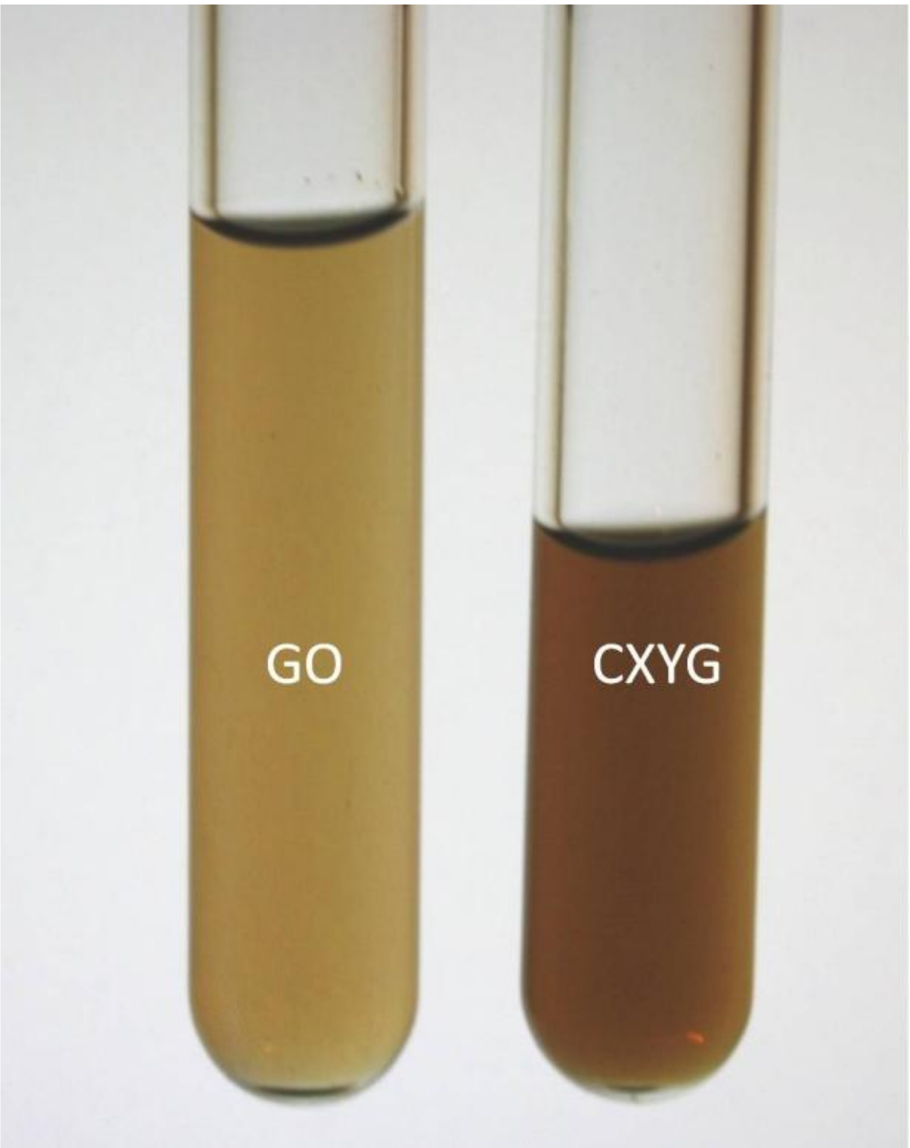

Figure 4: Photograph of GO and CXYG stock suspensions after 8 weeks of storage at 4 °C. GO and CXYG stock suspensions (160 and 320 µg/ml, respectively) demonstrated high colloidal stability. No sedimentation of GO or GXYG could be observed.

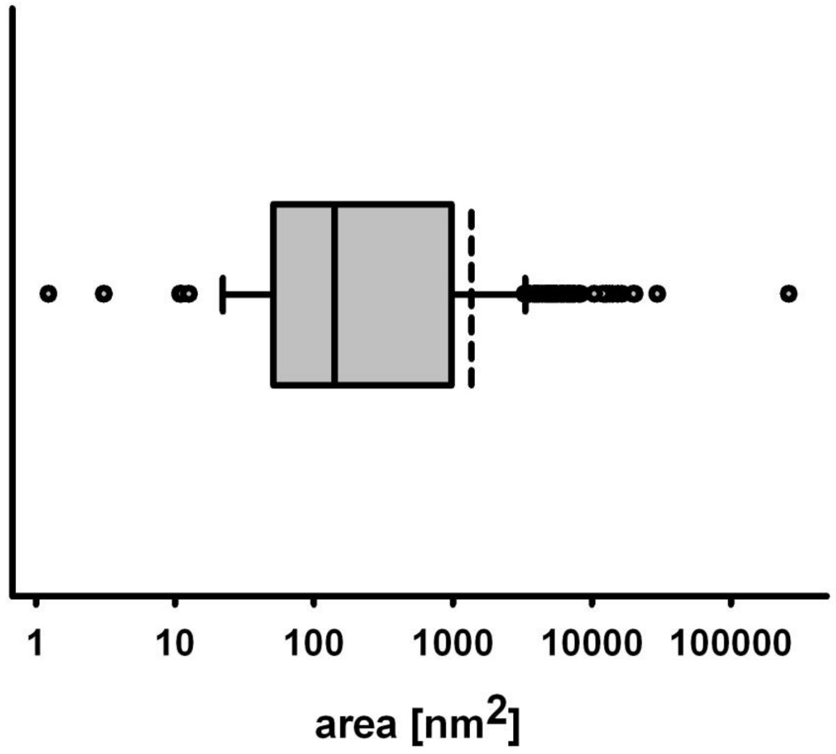

Figure 5: Size distribution of GO platelets in the stock suspension established on the basis of surface area measurements performed on AFM topographical images.

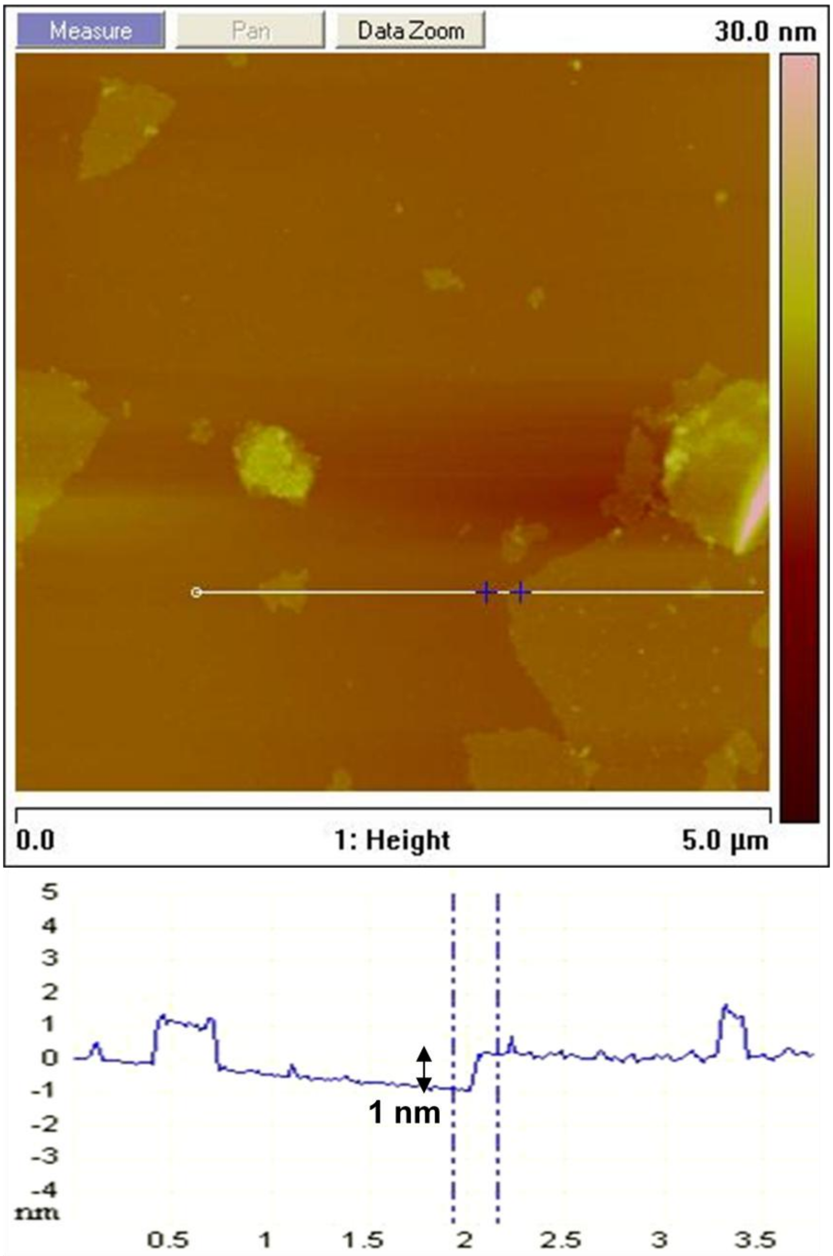

Figure 6: AFM topographical image of the GO stock solution. In addition to GO nanoplatelets with lateral dimensions in the lower nanometer range (cp. results section, Figure 2a), few GO platelets with lateral dimensions ranging from several hundreds of nanometers to a few micrometers could be identified.
