# Supplementary material for: Internalization and cytotoxicity of graphene oxide and carboxyl graphene nanoplatelets in the human hepatocellular carcinoma cell line Hep G2
Source: Part Fibre Toxicol. 2013 Jul 12;10:27. doi: 10.1186/1743-8977-10-27 (PMC3734190; doi:10.1186/1743-8977-10-27)
Supplement: Additional file 2: Figure S1 — Influence of culture medium composition on colloidal stability of GO and CXYG nanoplatelets. GO and CXYG stock suspensions were diluted 1:10 in three different complex cell culture media (A and B, respectively): MEM, MEM supplemented with 1% L-Gln and 1% P/S, and MEM supplemented with 1% L-Gln, 1% P/S and 10% FBS. The photographs were taken 10 minutes after preparation of the samples. Medium supplementation with FBS was essential to obtain dispersion with high colloidal stability. The presence of L-Gln and P/S did accelerate GO and CXYG nanoplatelet flocculation and sedimentation. Figure S2. Hydrodynamic size distribution in GO suspensions as function of concentration and incubation time. DLS analysis was performed on serial dilutions of a GO suspensions prepared in serum-supplemented culture medium (16 μg/ml). The samples were analyzed directly after preparation and after incubation at 37°C for 48 and 120 h, respectively. No significant change in the size distribution profile was observed as function of sample concentration or incubation time. Figure S3. Hydrodynamic size distribution in CXYG suspensions as function of concentration and incubation time. DLS analysis was performed on serial dilutions of a CXYG dispersion prepared in serum-supplemented culture medium (32 μg/ml). The samples were analyzed directly after preparation and after incubation at 37°C for 48 and 120 h, respectively. No significant change in the size distribution profile was observed as function of sample concentration or incubation time. Figure S4. Hydrodynamic size distribution profile of serum-supplemented MEM. [file 1743-8977-10-27-S2.pdf]

Additional file 2

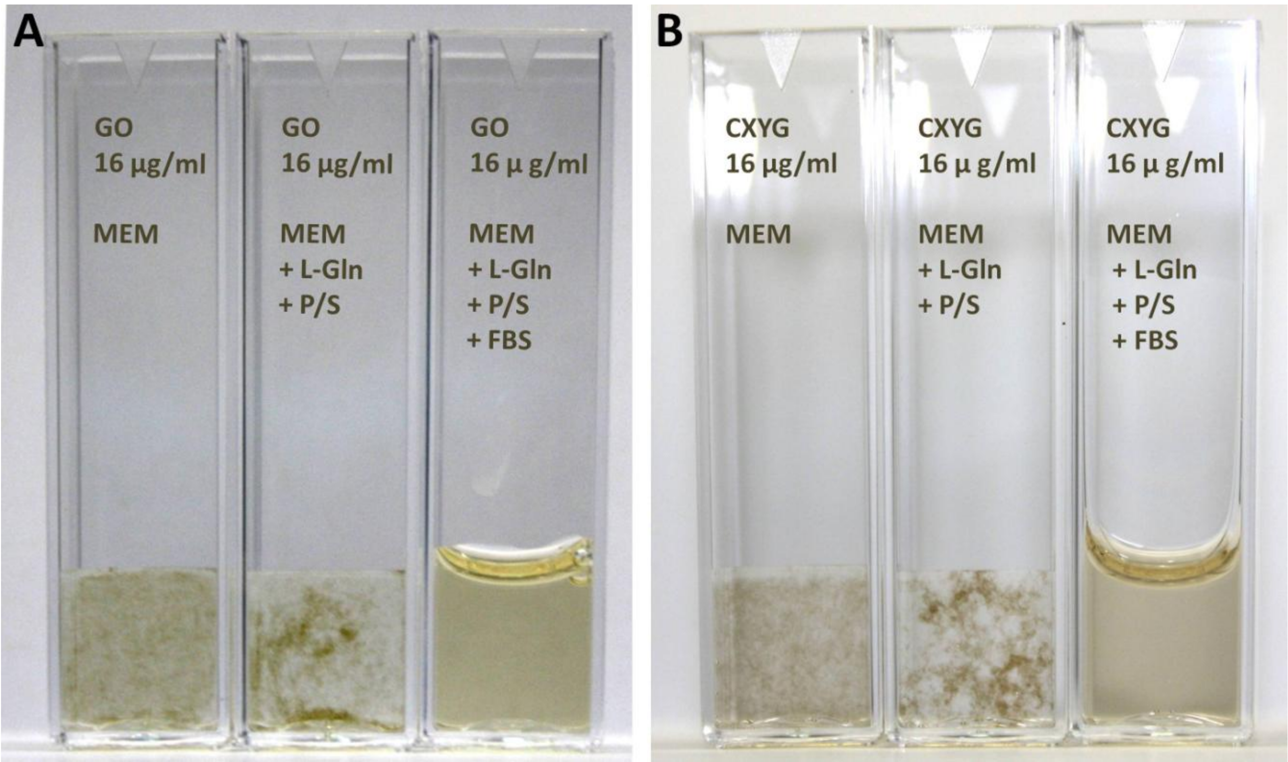

Figure 1: Influence of culture medium composition on colloidal stability of GO and CXYG nanoplatelets. GO and CXYG stock suspensions were diluted 1:10 in three different complex cell culture media (A and B, respectively): MEM, MEM supplemented with 1 % L-Gln and 1 % P/S, and MEM supplemented with 1 % L-Gln, 1 % P/S and 10 % FBS. The photographs were taken 10 minutes after preparation of the samples. Medium supplementation with FBS was essential to obtain dispersion with high colloidal stability. The presence of L-Gln and P/S did accelerate GO and CXYG nanoplatelet flocculation and sedimentation.

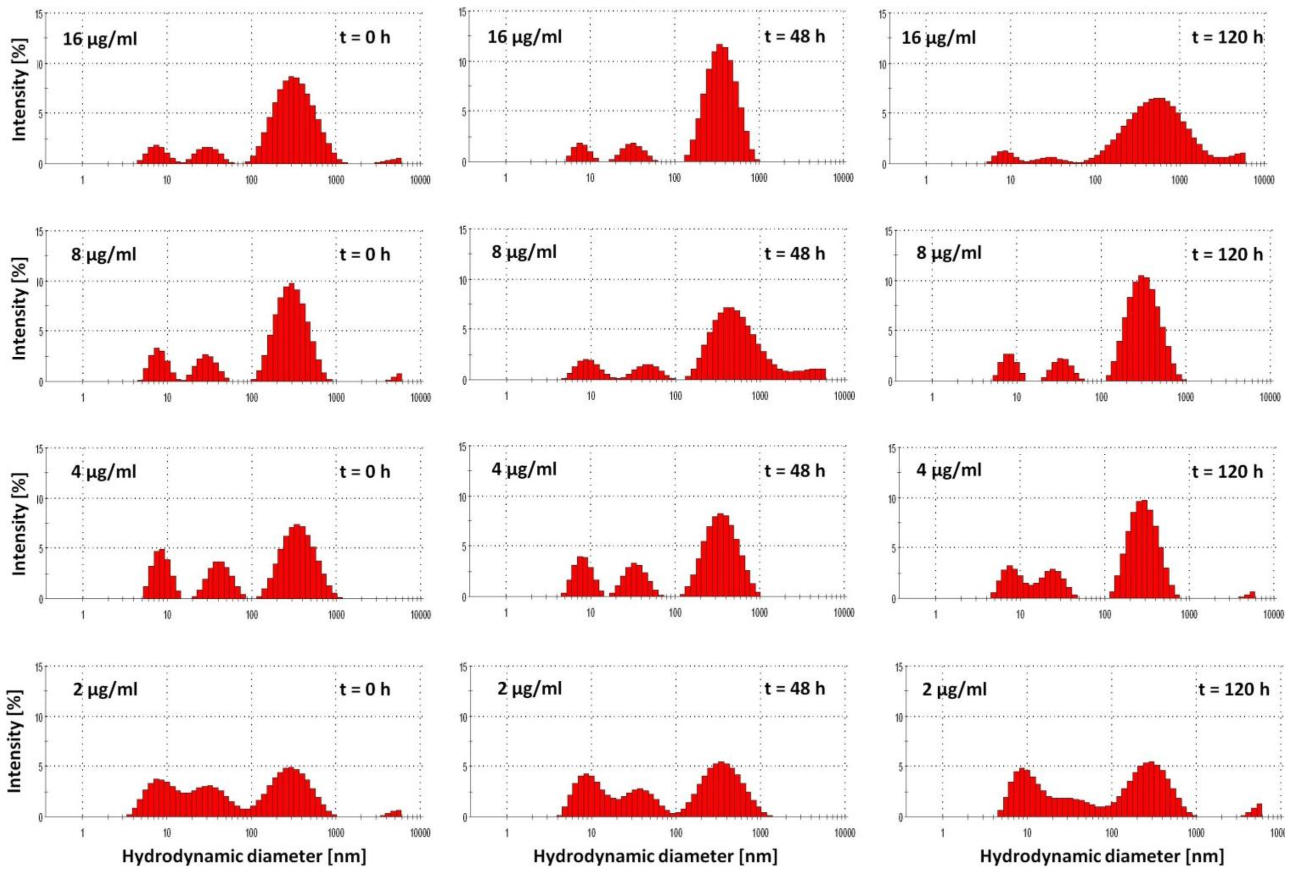

Figure 2: Hydrodynamic size distribution in GO suspensions as function of concentration and incubation time. DLS analysis was performed on serial dilutions of a GO suspensions prepared in serum-supplemented culture medium (16 µg/ml). The samples were analyzed directly after preparation and after incubation at 37 ºC for 48 and 120 h, respectively. No significant change in the size distribution profile was observed as function of sample concentration or incubation time.

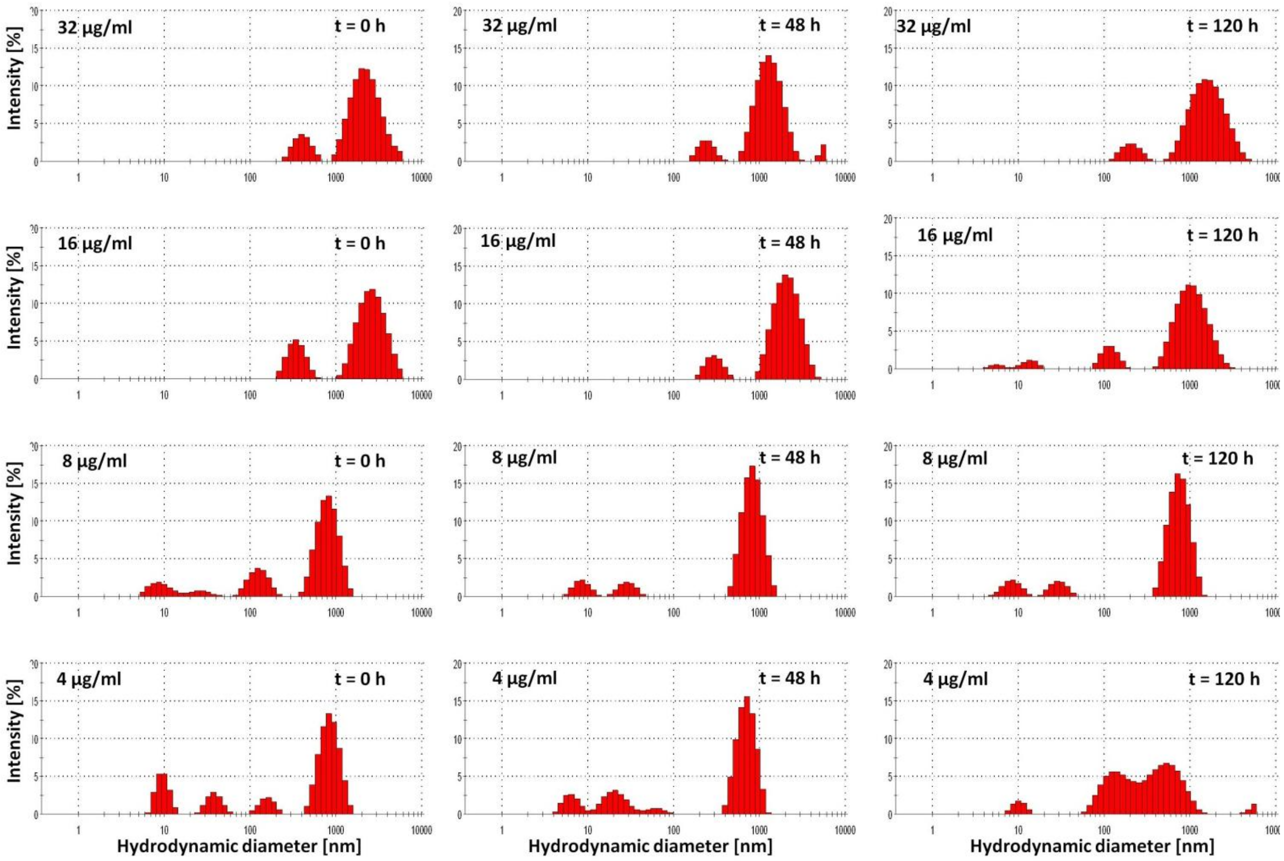

Figure 3: Hydrodynamic size distribution in CXYG suspensions as function of concentration and incubation time. DLS analysis was performed on serial dilutions of a CXYG dispersion prepared in serum-supplemented culture medium (32 µg/ml). The samples were analyzed directly after preparation and after incubation at 37 ºC for 48 and 120 h, respectively. No significant change in the size distribution profile was observed as function of sample concentration or incubation time.

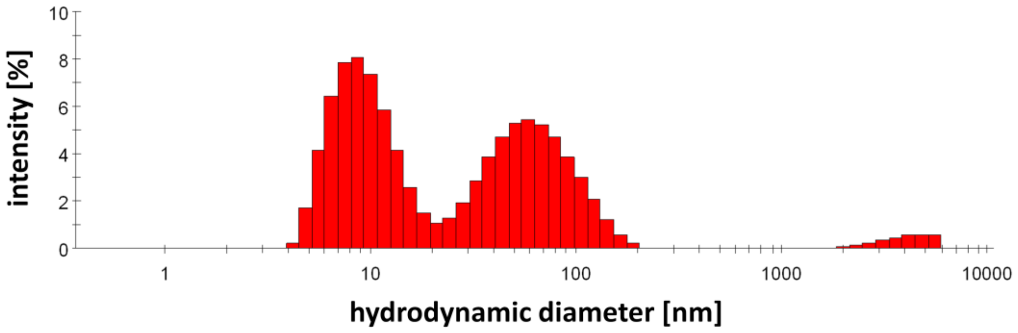

Figure 4: Hydrodynamic size distribution profile of serum-supplemented MEM.
