# Supplementary material for: Internalization and cytotoxicity of graphene oxide and carboxyl graphene nanoplatelets in the human hepatocellular carcinoma cell line Hep G2
Source: Part Fibre Toxicol. 2013 Jul 12;10:27. doi: 10.1186/1743-8977-10-27 (PMC3734190; doi:10.1186/1743-8977-10-27)
Supplement: Additional file 3: Figure S1 — Three-dimensional (3D) fluorescence spectra of GO and CXYG stock suspensions. A) 3D fluorescence spectrum of a 10 μg/ml GO/Milli-Q water dispersion B) 3D fluorescence spectrum of a 10 μg/ml CXYG/Milli-Q water dispersions. C) 3D fluorescence spectrum of Milli-Q water (blank). Figure S2. Redox-reaction of alamarBlue with GO and CXYG in absence of cells. AlamarBlue dissolved in phenol red-free medium without FBS was incubated with inreasing concentrations of GO and CXYG (0.2 – 100 μg/ml) at 37°C for 30 min. No reduction of alamarBlue was observed. [file 1743-8977-10-27-S3.pdf]

Additional file 3

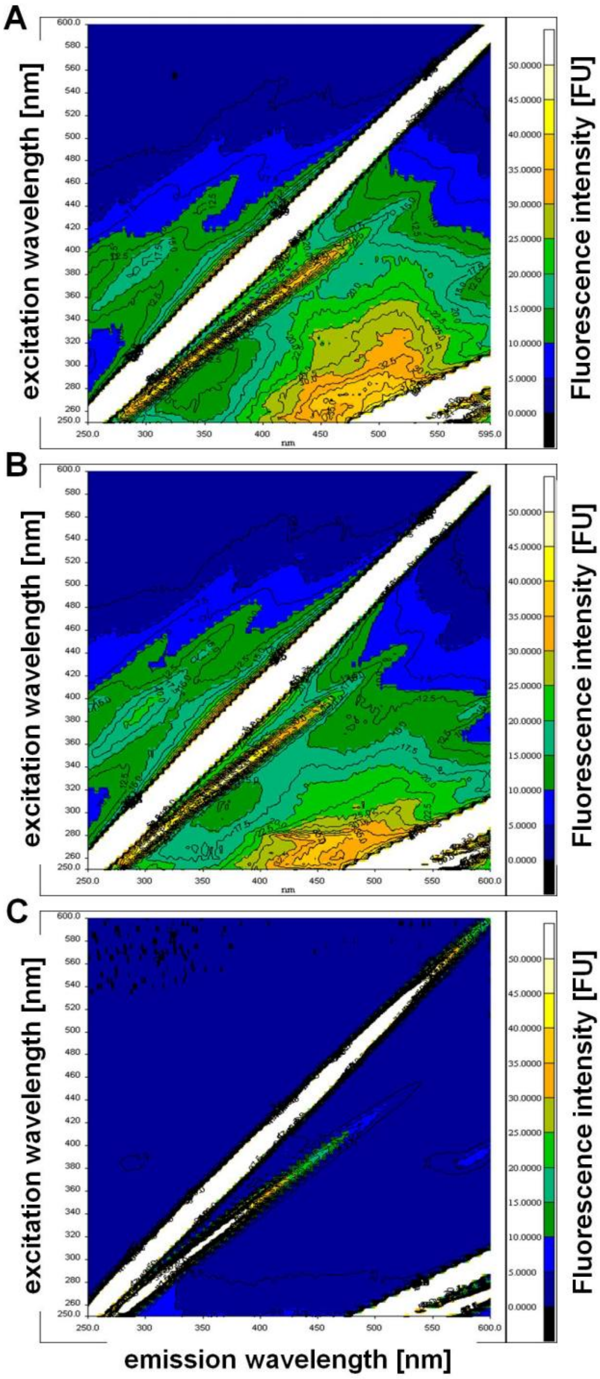

Figure 1: Three-dimensional (3D) fluorescence spectra of GO and CXYG stock suspensions. A) 3D fluorescence spectrum of a 10 µg/ml GO/Milli-Q water dispersion B) 3D fluorescence spectrum of a 10 µg/ml CXYG/Milli-Q water dispersions. C) 3D fluorescence spectrum of Milli-Q water (blank).

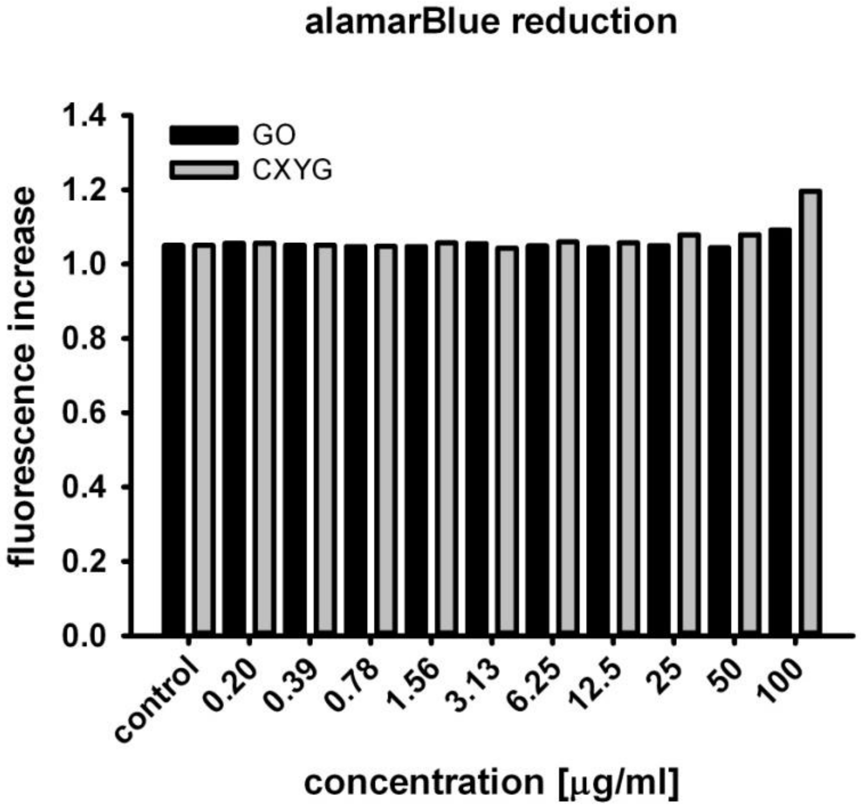

Figure 2: Redox-reaction of alamarBlue with GO and CXYG in absence of cells. AlamarBlue dissolved in phenol red-free medium without FBS was incubated with inreasing concentrations of GO and CXYG (0.2 – 100 µg/ml) at 37 ºC for 30 min. No reduction of alamarBlue was observed.
