# Supplementary material for: Internalization and cytotoxicity of graphene oxide and carboxyl graphene nanoplatelets in the human hepatocellular carcinoma cell line Hep G2
Source: Part Fibre Toxicol. 2013 Jul 12;10:27. doi: 10.1186/1743-8977-10-27 (PMC3734190; doi:10.1186/1743-8977-10-27)
Supplement: Additional file 4: Figure S1 — SEM micrographs of Hep G2 cells exposed to 16 and 32 μg/ml GO and CXYG for 72. Image A shows a SEM micrograph of untreated cells. Image B shows cells treated with 32 μg/ml CXYG. Image C shows cells treated with 16 μg/ml GO. The boxed-in area is shown at higher magnification in image D. Scale bares are 30 μm in A and B, 50 μm in C and 4 μm in D. White arrows exemplarily indicate apoptotic cells (communication with Dr. Covadonga Alonso, Departamento de Biotecnología, Instituto Nacional de Investigación y Tecnología Agraria y Alimentaria, INIA) being detached from the substrate and neighboring cells, and characterized by a round cell shape and plasma membrane blebbing. Figure S2. Differential cytotoxicity of GO and CXYG. [file 1743-8977-10-27-S4.pdf]

Additional file 4

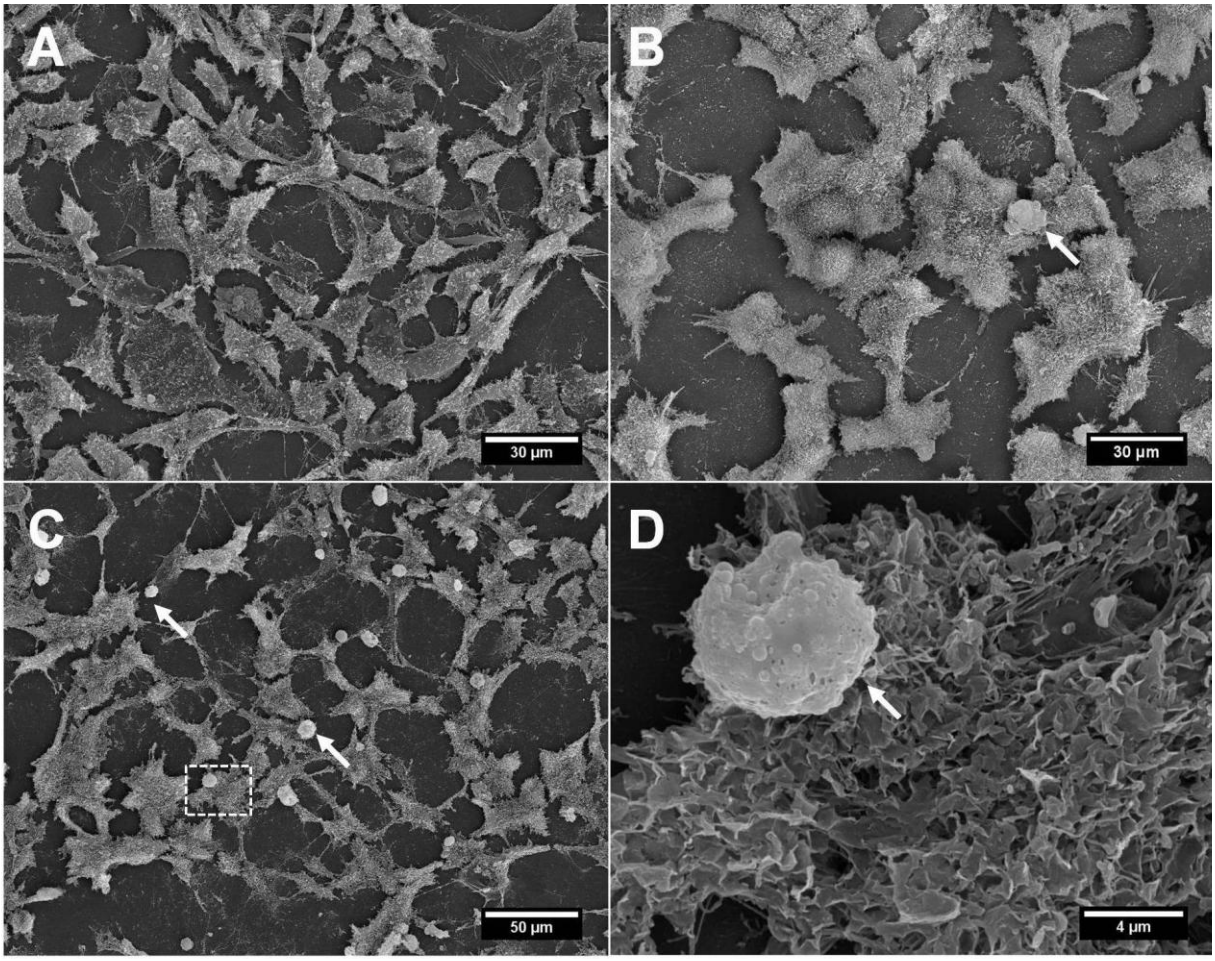

Figure 1: SEM micrographs of Hep G2 cells exposed to 16 and 32 µg/ml GO and CXYG for 72. Image A shows a SEM micrograph of untreated cells. Image B shows cells treated with 32 µg/ml CXYG. Image C shows cells treated with 16 µg/ml GO. The boxed-in area is shown at higher magnification in image D. Scale bares are 30 µm in A and B, 50 µm in C and 4 µm in D. White arrows exemplarily indicate apoptotic cells (communication with Dr. Covadonga Alonso, Departamento de Biotecnología, Instituto Nacional de Investigación y Tecnología Agraria y Alimentaria, INIA) being detached from the substrate and neighboring cells, and characterized by a round cell shape and plasma membrane blebbing.

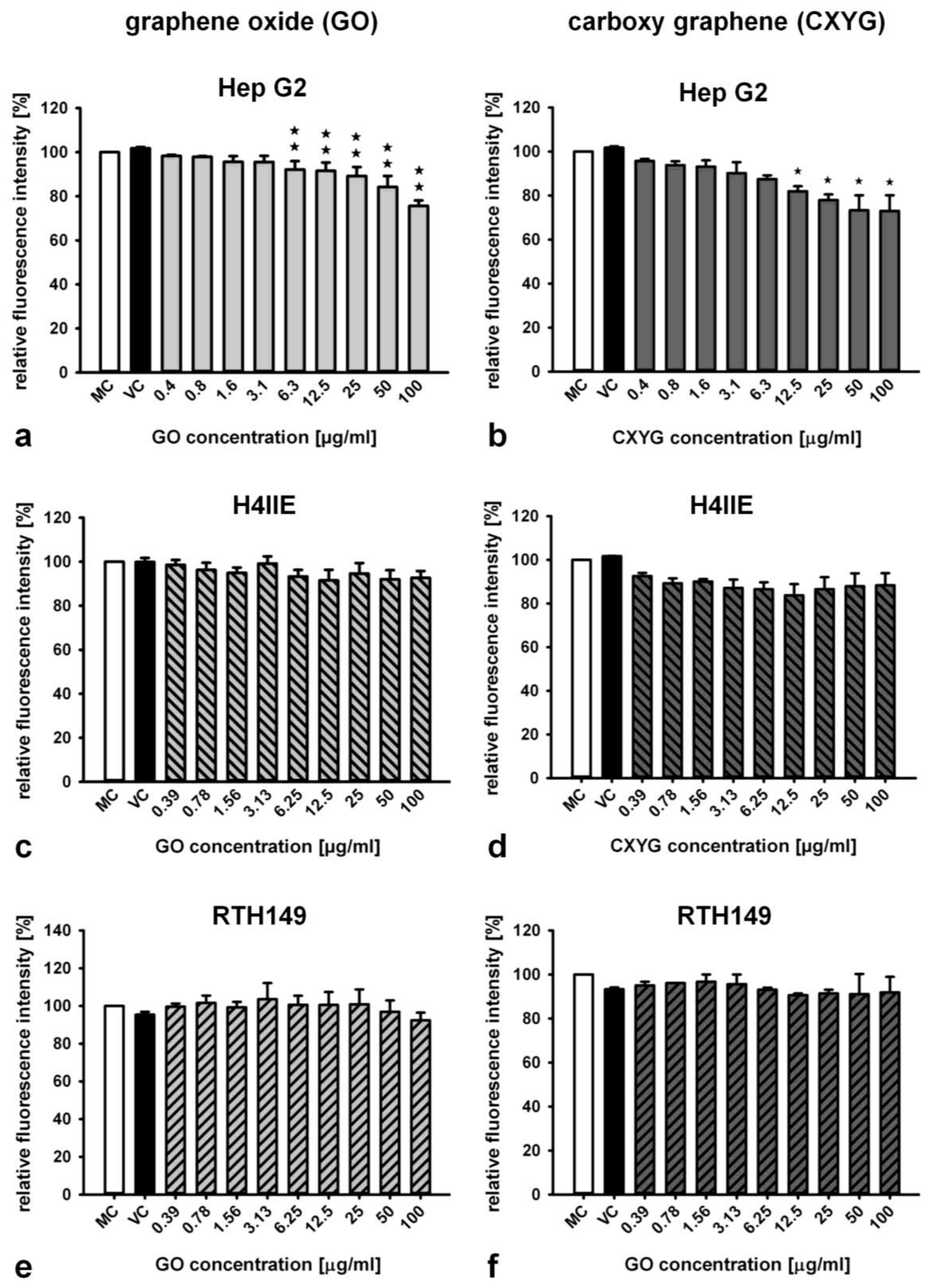

Figure 2: Differential cytotoxicity of GO and CXYG.
